# Supplementary material for: Genetic determinants of endophytism in the Arabidopsis root mycobiome
Source: Nat Commun. 2021 Dec 10;12:7227. doi: 10.1038/s41467-021-27479-y (PMC8664821; doi:10.1038/s41467-021-27479-y)
Supplement: Supplementary file 1 — Supplementary Information [file 41467_2021_27479_MOESM1_ESM.pdf]

Supplementary information

Genetic determinants of endophytism in the *Arabidopsis* root mycobiome

Fantin Mesny[1], Shingo Miyauchi[1,2], Thorsten Thiergart[1], Brigitte Pickel[1], Lea Atanasova[3,4], Magnus Karlsson[5], Bruno Hüttel[6], Kerrie W. Barry[7], Sajeet Haridas[7], Cindy Chen[7], Diane Bauer[7], William Andreopoulos[7], Jasmyn Pangilinan[7], Kurt LaButti[7], Robert Riley[7], Anna Lipzen[7], Alicia Clum[7], Elodie Drula[8,9], Bernard Henrissat[10], Annegret Kohler[2], Igor V. Grigoriev[7,11], Francis M. Martin[2,12,*], Stéphane Hacquard[1,13,*].

[1]Max Planck Institute for Plant Breeding Research, 50829 Cologne, Germany. [2]Université de Lorraine, Institut national de recherche pour l'agriculture, l'alimentation et l'environnement, UMR Interactions Arbres/Microorganismes, Centre INRAE Grand Est-Nancy, 54280, Champenoux, France. [3]Research division of Biochemical Technology, Institute of Chemical, Environmental and Biological Engineering, Vienna University of Technology, Vienna, Austria. [4]Institute of Food Technology, University of Natural Resources and Life Sciences, Vienna, Austria. [5]Forest Mycology and Plant Pathology, Swedish University of Agricultural Sciences, SE-75007, Uppsala, Sweden. [6]Max Planck Genome Centre Cologne, Max Planck Institute for Plant Breeding Research, Cologne, Germany. [7]U.S. Department of Energy Joint Genome Institute, Lawrence Berkeley National Laboratory, Berkeley, CA, USA. [8]INRAE, USC1408 Architecture et Fonction des Macromolécules Biologiques, 13009, Marseille, France. [9]Architecture et Fonction des Macromolécules Biologiques (AFMB), CNRS, Aix-Marseille Univ., 13009, Marseille, France. [10]Department of Biological Sciences, King Abdulaziz University, Jeddah, Saudi Arabia. [11]Department of Plant and Microbial Biology, University of California Berkeley, Berkeley, CA, USA. [12]Beijing Advanced Innovation Centre for Tree Breeding by Molecular Design (BAIC-TBMD), Institute of Microbiology, Beijing Forestry University, Tsinghua East Road Haidian District, Beijing, China. [13]Cluster of Excellence on Plant Sciences (CEPLAS), Max Planck Institute for Plant Breeding Research, 50829 Cologne, Germany.

*Corresponding authors: F. M. Martin (francis.martin@inrae.fr) and S. Hacquard (hacquard@mpipz.mpg.de).

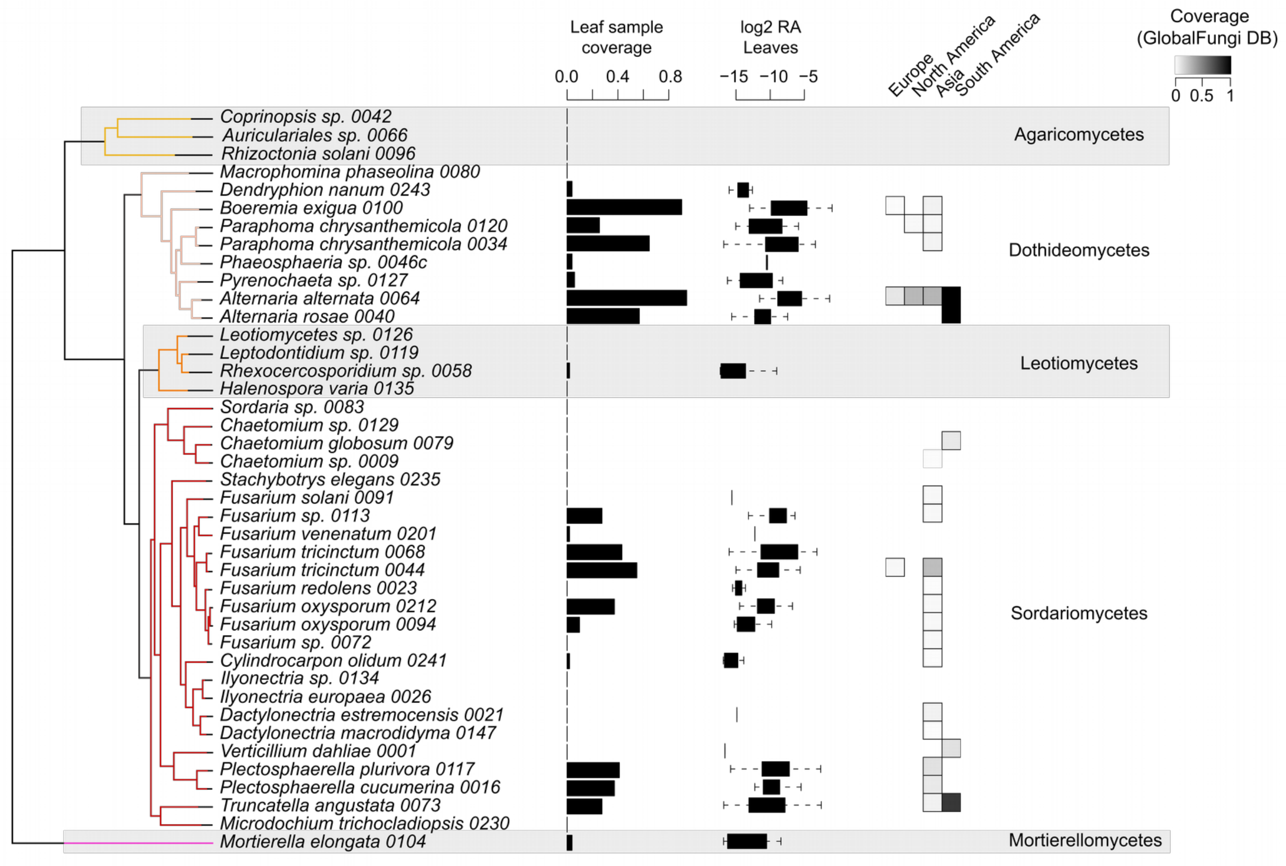

Supplementary figure 1: Prevalence and abundance profiles of 41 root-colonizing fungi across naturally occurring *A. thaliana* shoot mycobiomes.
Relative abundance and sample coverage across leaf samples taken from wild *A. thaliana* plants harvested at two locations in Germany (Cologne and Tübingen[39]). Fungal rDNA ITS2 sequences were directly mapped to raw sequencing reads with a 97% similarity threshold. Leaf sample coverage refers to the percentage of leaf samples with relative abundances superior to 0.01% (leaf samples n=51). On relative abundance boxplots, boxes are delimited by first and third quartiles and whiskers extend to minimum and maximum values. Unmapped reads were used to estimate relative abundance of other fungal species. The coverage of global shoot samples was estimated by checking the occurrence of rDNA ITS1 sequences in 2,647 shoot samples (Asia n=250, Europe n= 1759, North America n= 602, South America n=36) retrieved from the GlobalFungi database[38].

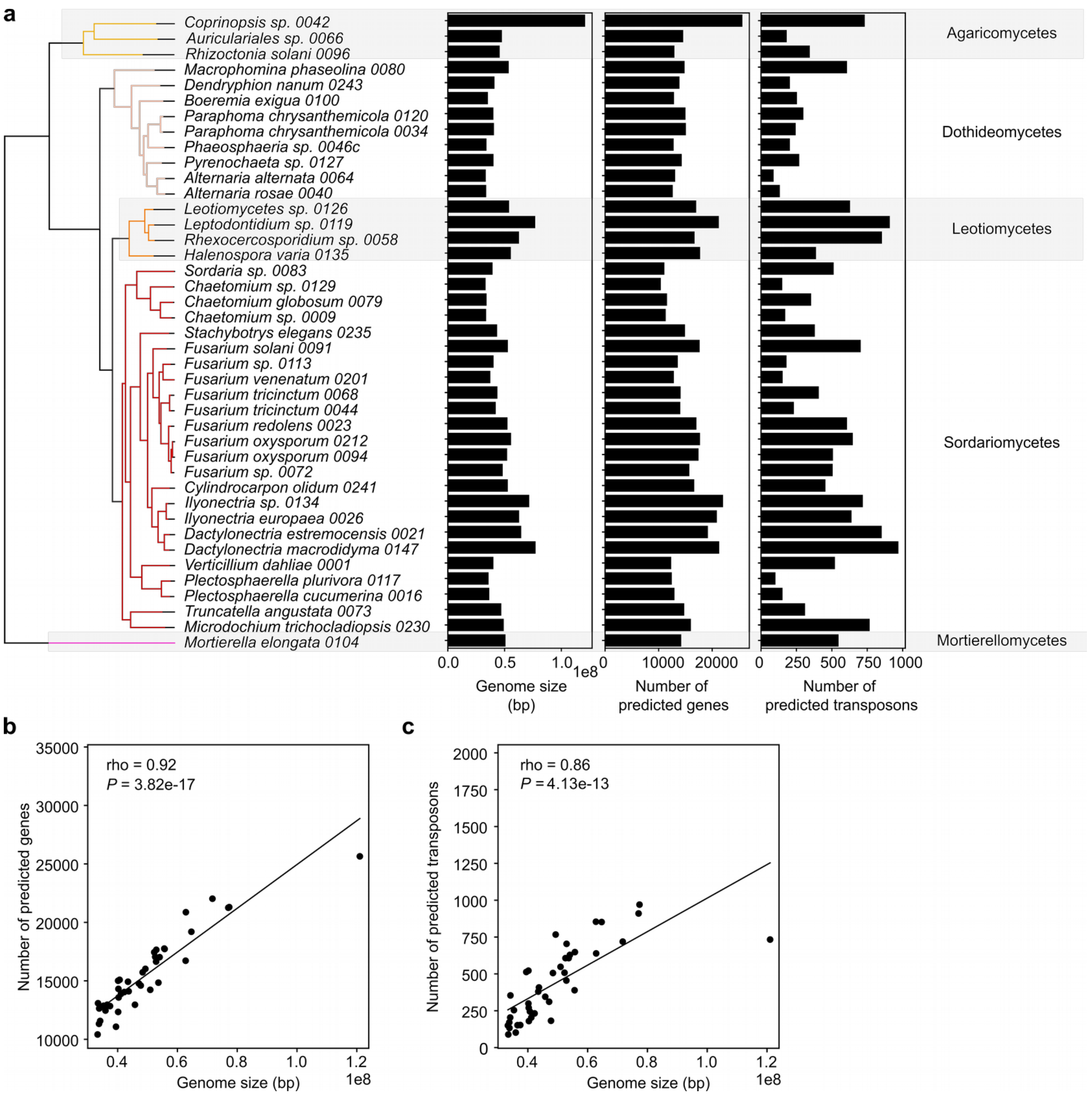

Supplementary figure 2: Link between genome size, number of genes and number of transposons across the 41 newly-sequenced fungal strains.

a, Genome assembly size, number of predicted genes and number of identified transposons in the genomes of the 41 *A. thaliana* mycobiota members. **b**, Spearman's rank correlation (rho, $P < 0.05$) between genome size and number of predicted genes. **c**, Spearman's rank correlation (rho, $P < 0.05$) between genome size and number of predicted transposable elements.

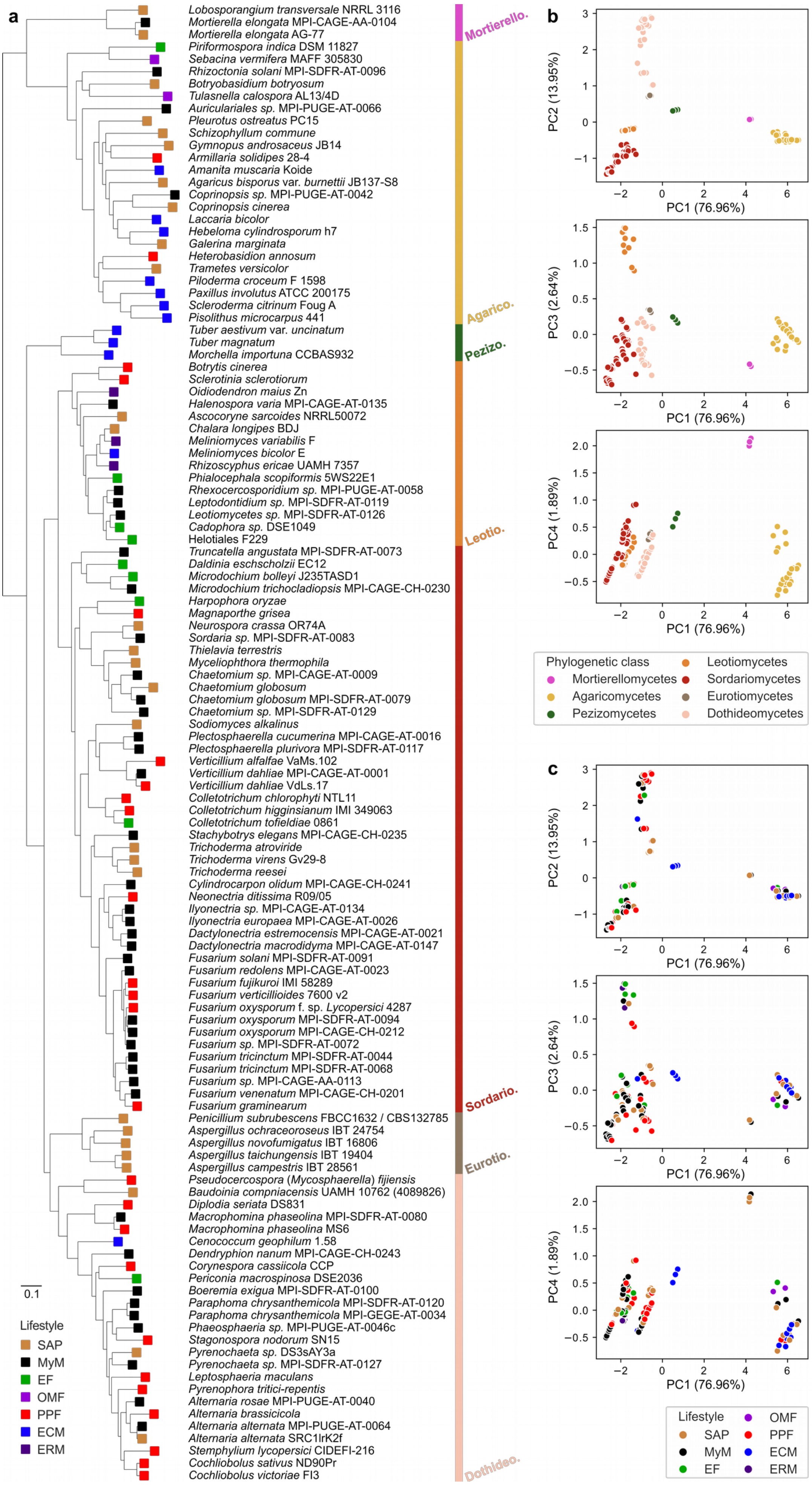
a
Lobosporangium transversale NRRL 3116
Mortierella elongata MPI-CAGE-AA-0104
Mortierella elongata AG-77
Piriformospora indica DSM 11827
Sebacina vermifera MAFF 305830
Rhizoctonia solani MPI-SDFR-AT-0096
Botryobasidium botryosum
Tulasnella calospora AL13/4D
Auriculariales sp. MPI-PUGE-AT-0066
Pleurotus ostreatus PC15
Schizophyllum commune
Gymnopus androsaceus JB14
Armillaria solidipes 28-4
Amanita muscaria Koide
Agaricus bisporus var. burnettii JB137-S8
Coprinopsis sp. MPI-PUGE-AT-0042
Coprinopsis cinerea
Laccaria bicolor
Hebeloma cylindrosporum h7
Galerina marginata
Heterobasidion annosum
Trametes versicolor
Piloderma croceum F 1598
Paxillus involutus ATCC 200175
Scleroderma citrinum Foug A
Pisolithus microcarpus 441
Tuber aestivum var. uncinatum
Tuber magnatum
Morchella importuna CCBAS932
Botrytis cinerea
Sclerotinia sclerotiorum
Oidiodendron maius Zn
Halenospora varia MPI-CAGE-AT-0135
Ascocoryne sarcoides NRRL50072
Chalara longipes BDJ
Meliniomyces variabilis F
Meliniomyces bicolor E
Rhizoscyphus ericae UAMH 7357
Phialocephala scopiformis 5WS22E1
Rhexocercosporidium sp. MPI-PUGE-AT-0058
Leptodontidium sp. MPI-SDFR-AT-0119
Leotiomycetes sp. MPI-SDFR-AT-0126
Cadophora sp. DSE1049
Helotiales F229
Truncatella angustata MPI-SDFR-AT-0073
Daldinia eschscholzii EC12
Microdochium bolleyi J235TASD1
Microdochium trichocladiopsis MPI-CAGE-CH-0230
Harpophora oryzae
Magnaporthe grisea
Neurospora crassa OR74A
Sordaria sp. MPI-SDFR-AT-0083
Thielavia terrestris
Myceliophthora thermophila
Chaetomium sp. MPI-CAGE-AT-0009
Chaetomium globosum
Chaetomium globosum MPI-SDFR-AT-0079
Chaetomium sp. MPI-SDFR-AT-0129
Sodiomyces alkalinus
Plectosphaerella cucumerina MPI-CAGE-AT-0016
Plectosphaerella plurivora MPI-SDFR-AT-0117
Verticillium alfalfae VaMs.102
Verticillium dahliae MPI-CAGE-AT-0001
Verticillium dahliae VdLs.17
Colletotrichum chlorophyti NTL11
Colletotrichum higginsianum IMI 349063
Colletotrichum tofieldiae 0861
Stachybotrys elegans MPI-CAGE-CH-0235
Trichoderma atroviride
Trichoderma virens Gv29-8
Trichoderma reesei
Cylindrocarpon olidum MPI-CAGE-CH-0241
Neonectria ditissima R09/05
Ilyonectria sp. MPI-CAGE-AT-0134
Ilyonectria europaea MPI-CAGE-AT-0026
Dactylonectria estremocensis MPI-CAGE-AT-0021
Dactylonectria macrodidyma MPI-CAGE-AT-0147
Fusarium solani MPI-SDFR-AT-0091
Fusarium redolens MPI-CAGE-AT-0023
Fusarium fujikuroi IMI 58289
Fusarium verticillioides 7600 v2
Fusarium oxysporum f. sp. Lycopersici 4287
Fusarium oxysporum MPI-SDFR-AT-0094
Fusarium oxysporum MPI-CAGE-CH-0212
Fusarium sp. MPI-SDFR-AT-0072
Fusarium tricinctum MPI-SDFR-AT-0044
Fusarium tricinctum MPI-SDFR-AT-0068
Fusarium sp. MPI-CAGE-AA-0113
Fusarium venenatum MPI-CAGE-CH-0201
Fusarium graminearum
Penicillium subrubescens FBCC1632 / CBS132785
Aspergillus ochraceoroseus IBT 24754
Aspergillus novofumigatus IBT 16806
Aspergillus taichungensis IBT 19404
Aspergillus campestris IBT 28561
Pseudocercospora (Mycosphaerella) fijiensis
Baudoinia compniacensis UAMH 10762 (4089826)
Diplodia seriata DS831
Macrophomina phaseolina MPI-SDFR-AT-0080
Macrophomina phaseolina MS6
Cenococcum geophilum 1.58
Dendryphion nanum MPI-CAGE-CH-0243
Corynespora cassiicola CCP
Periconia macrospinosa DSE2036
Boeremia exigua MPI-SDFR-AT-0100
Paraphoma chrysanthemicola MPI-SDFR-AT-0120
Paraphoma chrysanthemicola MPI-GEGE-AT-0034
Phaeosphaeria sp. MPI-PUGE-AT-0046c
Stagonospora nodorum SN15
Pyrenochaeta sp. DS3sAY3a
Pyrenochaeta sp. MPI-SDFR-AT-0127
Leptosphaeria maculans
Pyrenophora tritici-repentis
Alternaria rosae MPI-PUGE-AT-0040
Alternaria brassicicola
Alternaria alternata MPI-PUGE-AT-0064
Alternaria alternata SRC1lrK2f
Stemphylium lycopersici CIDEFI-216
Cochliobolus sativus ND90Pr
Cochliobolus victoriae FI3
Mortierello.
Agarico.
Pezizo.
Leotio.
Sordario.
Eurotio.
Dothideo.
0.1
Lifestyle
SAP
MyM
EF
OMF
PPF
ECM
ERM
b
PC2 (13.95%)
PC3 (2.64%)
PC4 (1.89%)
PC1 (76.96%)
Phylogenetic class
Mortierellomycetes
Agaricomycetes
Pezizomycetes
Leotiomycetes
Sordariomycetes
Eurotiomycetes
Dothideomycetes
c
PC2 (13.95%)
PC3 (2.64%)
PC4 (1.89%)
PC1 (76.96%)
Lifestyle
SAP
MyM
EF
OMF
PPF
ECM
ERM

Supplementary figure 3: Phylogeny of the 120-genome data set used for comparative genomics.
a, Species tree describing the phylogeny of the 120 fungal genomes used for comparative genomics. This tree was inferred from full sets of proteins by OrthoFinder[46] after orthology prediction. Leaf tip colors represent fungal lifestyles, and color strips on the right highlight the different phylogenetic classes. **bc**, Principal component analysis (PCA) calculated on phylogenetic pairwise distances extracted from the aforementioned species tree. Panels **b** and **c** represent the same PCA plot with different colors representing either the phylogenetic class (**b**) or the fungal lifestyle (**c**).
SAP: Saprotrophs, MyM: *A. thaliana* mycobiota members, EF: Endophytic Fungi, OMF: Orchid Mycorrhizal Fungi, PPF: Plant Pathogenic Fungi, ECM: Ectomycorrhiza, ERM: Ericoid Mycorrhiza.

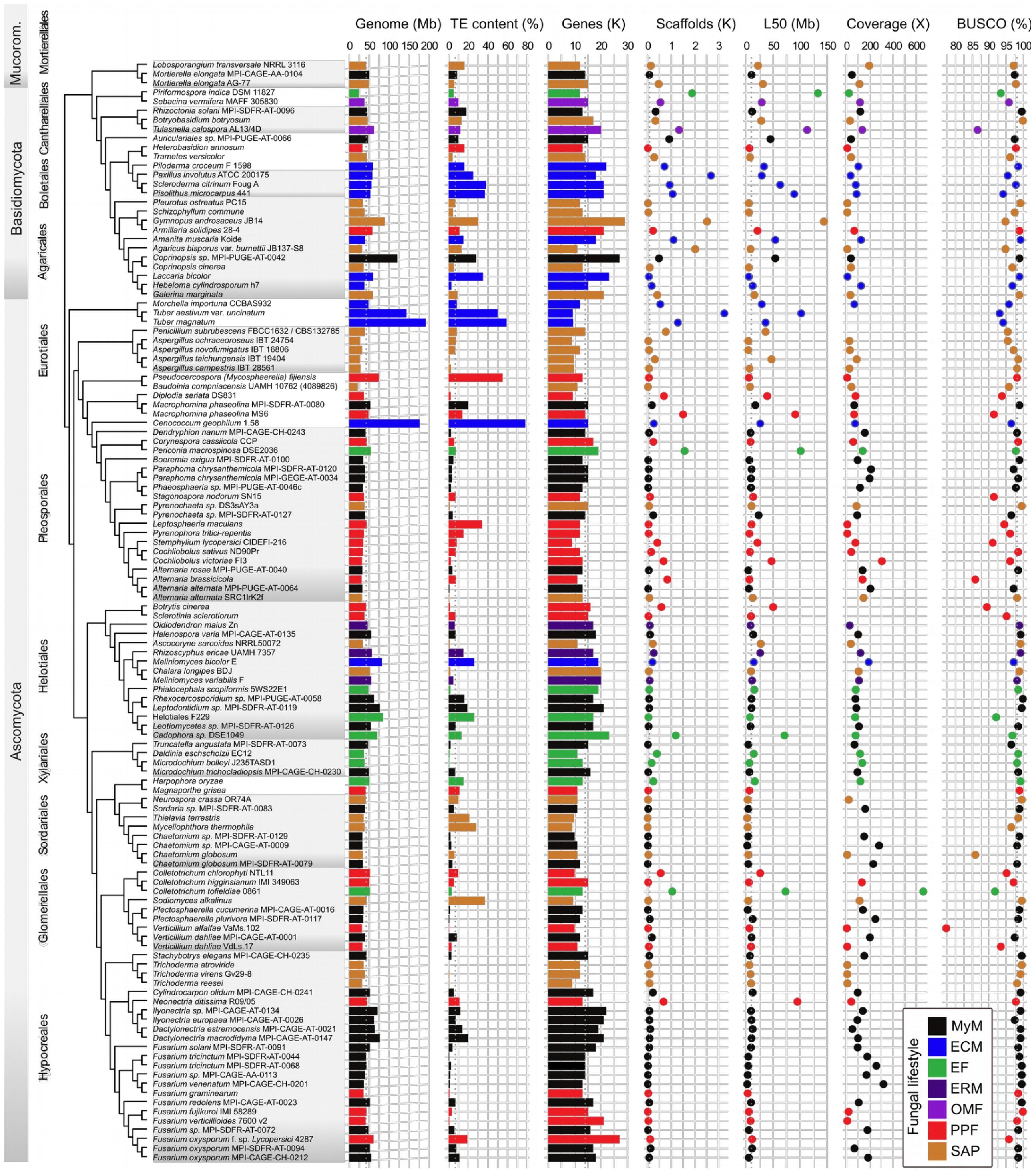

Supplementary figure 4: Genome sizes and properties of the 41 root mycobiota members, along with 79 previously published genomes used for comparative genomics.

Fungal lifestyle is depicted in color. Median values are marked with dotted line. Genome: genome size. TE content: the coverage of transposable elements in the genomes. Genes: the number of genes. Secreted: the number of predicted secreted proteins (**Methods**). Scaffolds: the number of scaffolds. L50: N50 length. Coverage: sequencing depth in fold. BUSCO: genome completeness. MyM: *A. thaliana* mycobiota members, ECM: Ectomycorrhiza, EF: Endophytic Fungi, ERM: Ericoid Mycorrhiza, OMF: Orchid Mycorrhizal Fungi, PPF: Plant Pathogenic Fungi, SAP: Saprotrophs.

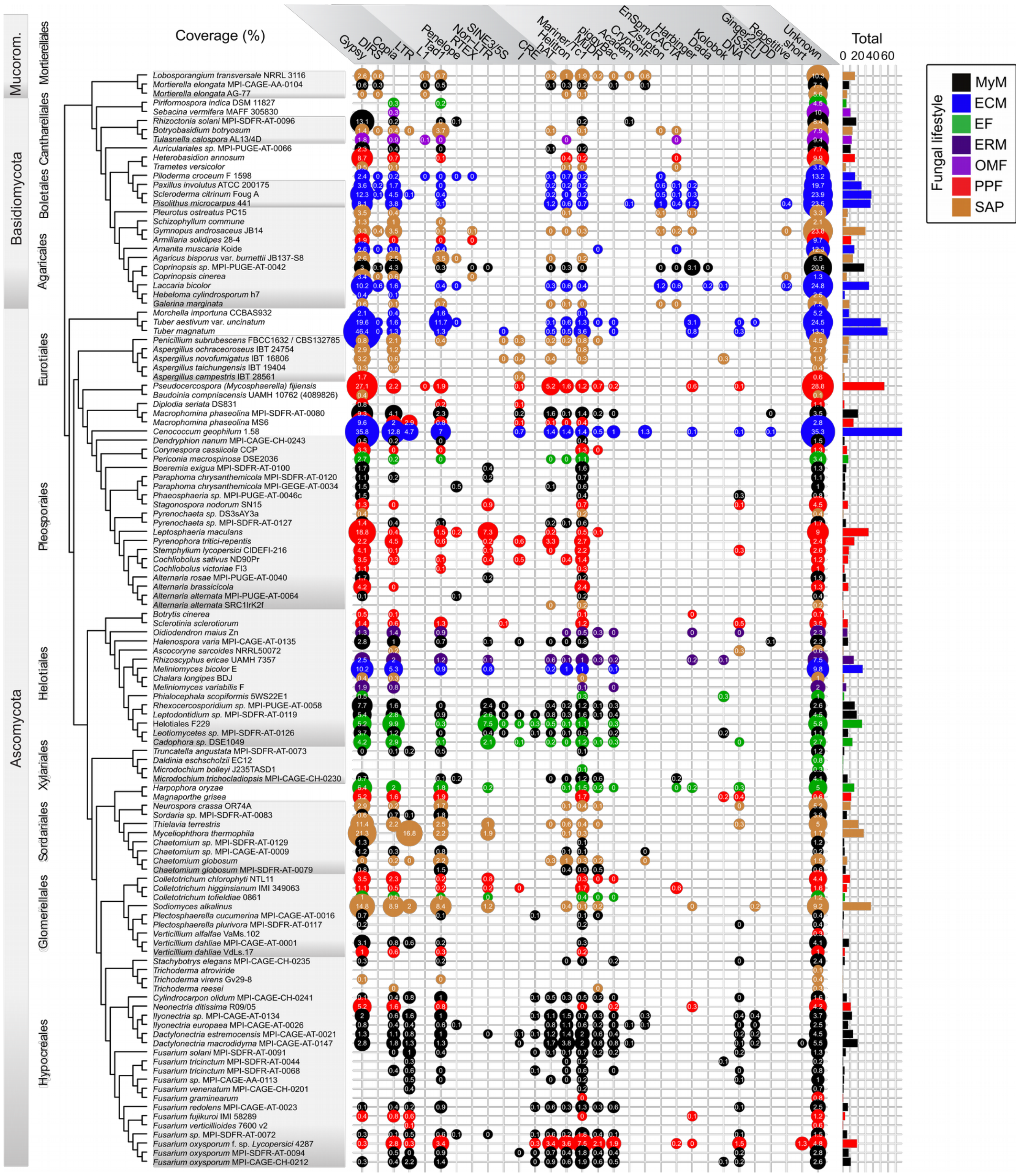

Supplementary figure 5: Compositions in transposable elements of the 120 fungal genomes used for comparative genomics.

Coverage of transposable elements in the 120 fungal genome dataset. LTR: long-terminal repeat retrotransposons. Non-LTR: non-long-terminal repeat retrotransposons. DNA: DNA transposons. Repetitive/short: simple repeats. Unknown: unclassified repeated sequences. The bubble size is proportional to the coverage of each of the transposable elements (shown inside the bubbles). The barplot on the right shows the total transposon coverage per genome. MyM: *A. thaliana* mycobiota members, ECM: Ectomycorrhiza, EF: Endophytic Fungi, ERM: Ericoid Mycorrhiza, OMF: Orchid Mycorrhizal Fungi, PPF: Plant Pathogenic Fungi, SAP: Saprotrophs.

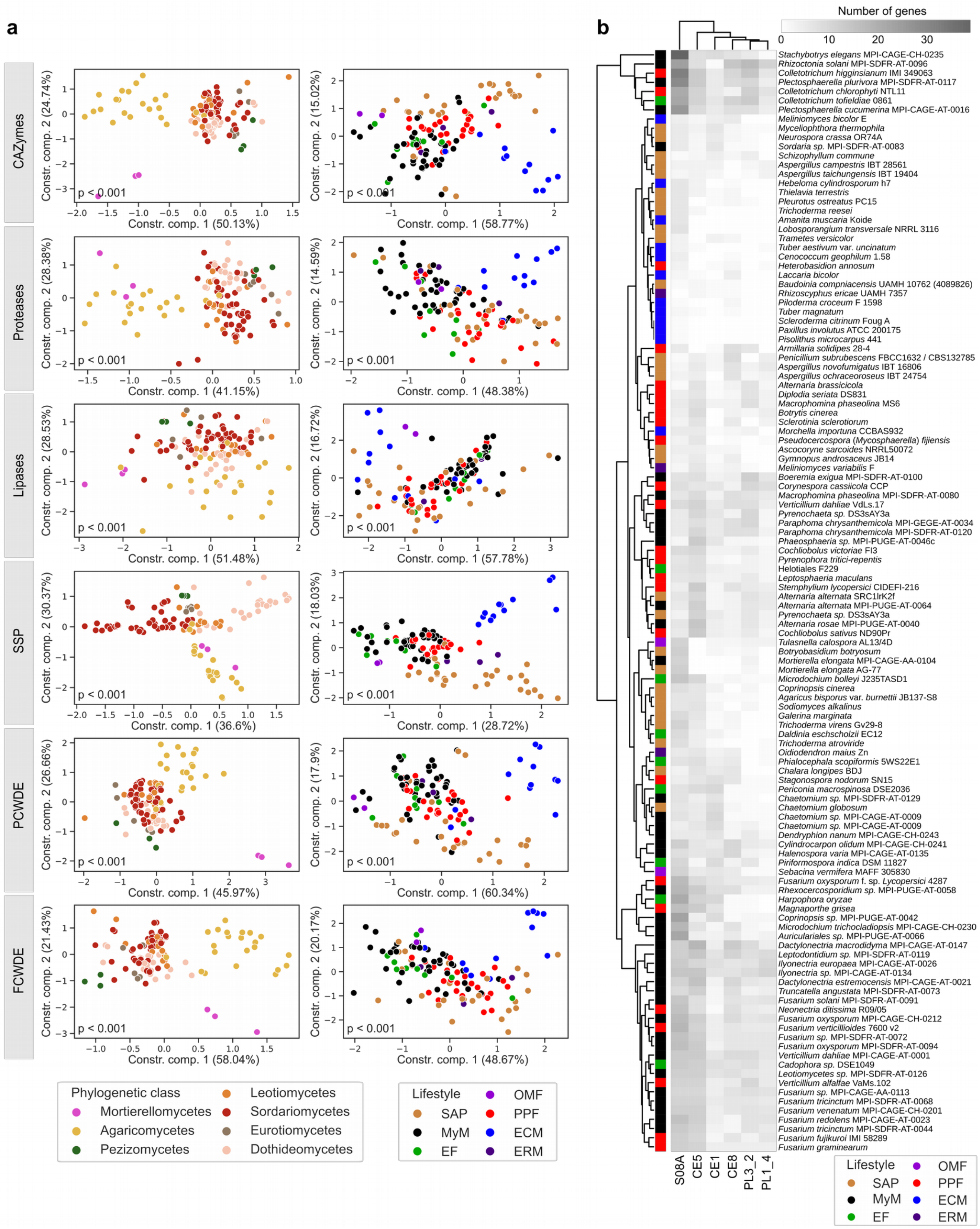
a
CAZymes
Constr. comp. 2 (24.74%)
Constr. comp. 1 (50.13%)
Constr. comp. 2 (15.02%)
Constr. comp. 1 (58.77%)
Proteases
Constr. comp. 2 (28.38%)
Constr. comp. 1 (41.15%)
Constr. comp. 2 (14.59%)
Constr. comp. 1 (48.38%)
Lipases
Constr. comp. 2 (28.53%)
Constr. comp. 1 (51.48%)
Constr. comp. 2 (16.72%)
Constr. comp. 1 (57.78%)
SSP
Constr. comp. 2 (30.37%)
Constr. comp. 1 (36.6%)
Constr. comp. 2 (18.03%)
Constr. comp. 1 (28.72%)
PCWDE
Constr. comp. 2 (26.66%)
Constr. comp. 1 (45.97%)
Constr. comp. 2 (17.9%)
Constr. comp. 1 (60.34%)
FCWDE
Constr. comp. 2 (21.43%)
Constr. comp. 1 (58.04%)
Constr. comp. 2 (20.17%)
Constr. comp. 1 (48.67%)
p < 0.001
Phylogenetic class
Mortierellomycetes
Agaricomycetes
Pezizomycetes
Leotiomycetes
Sordariomycetes
Eurotiomycetes
Dothideomycetes
Lifestyle
SAP
MyM
EF
OMF
PPF
ECM
ERM
b
Number of genes
0 10 20 30
Stachybotrys elegans MPI-CAGE-CH-0235
Rhizoctonia solani MPI-SDFR-AT-0096
Colletotrichum higginsianum IMI 349063
Plectosphaerella plurivora MPI-SDFR-AT-0117
Colletotrichum chlorophyti NTL11
Colletotrichum tofieldiae 0861
Plectosphaerella cucumerina MPI-CAGE-AT-0016
Meliniomyces bicolor E
Myceliophthora thermophila
Neurospora crassa OR74A
Sordaria sp. MPI-SDFR-AT-0083
Schizophyllum commune
Aspergillus campestris IBT 28561
Aspergillus taichungensis IBT 19404
Hebeloma cylindrosporum h7
Thielavia terrestris
Pleurotus ostreatus PC15
Trichoderma reesei
Amanita muscaria Koide
Lobosporangium transversale NRRL 3116
Trametes versicolor
Tuber aestivum var. uncinatum
Cenococcum geophilum 1.58
Heterobasidion annosum
Laccaria bicolor
Baudoinia compniacensis UAMH 10762 (4089826)
Rhizoscyphus ericae UAMH 7357
Piloderma croceum F 1598
Tuber magnatum
Scleroderma citrinum Foug A
Paxillus involutus ATCC 200175
Pisolithus microcarpus 441
Armillaria solidipes 28-4
Penicillium subrubescens FBCC1632 / CBS132785
Aspergillus novofumigatus IBT 16806
Aspergillus ochraceoroseus IBT 24754
Alternaria brassicicola
Diplodia seriata DS831
Macrophomina phaseolina MS6
Botrytis cinerea
Sclerotinia sclerotiorum
Morchella importuna CCBAS932
Pseudocercospora (Mycosphaerella) fijiensis
Ascocoryne sarcoides NRRL50072
Gymnopus androsaceus JB14
Meliniomyces variabilis F
Boeremia exigua MPI-SDFR-AT-0100
Corynespora cassiicola CCP
Macrophomina phaseolina MPI-SDFR-AT-0080
Verticillium dahliae VdLs.17
Pyrenochaeta sp. DS3sAY3a
Paraphoma chrysanthemicola MPI-GEGE-AT-0034
Paraphoma chrysanthemicola MPI-SDFR-AT-0120
Phaeosphaeria sp. MPI-PUGE-AT-0046c
Cochliobolus victoriae FI3
Pyrenophora tritici-repentis
Helotiales F229
Leptosphaeria maculans
Stemphylium lycopersici CIDEFI-216
Alternaria alternata SRC1lrK2f
Alternaria alternata MPI-PUGE-AT-0064
Pyrenochaeta sp. DS3sAY3a
Alternaria rosae MPI-PUGE-AT-0040
Cochliobolus sativus ND90Pr
Tulasnella calospora AL13/4D
Botryobasidium botryosum
Mortierella elongata MPI-CAGE-AA-0104
Mortierella elongata AG-77
Microdochium bolleyi J235TASD1
Coprinopsis cinerea
Agaricus bisporus var. burnettii JB137-S8
Sodiomyces alkalinus
Galerina marginata
Trichoderma virens Gv29-8
Daldinia eschscholzii EC12
Trichoderma atroviride
Oidiodendron maius Zn
Phialocephala scopiformis 5WS22E1
Chalara longipes BDJ
Stagonospora nodorum SN15
Periconia macrospinosa DSE2036
Chaetomium sp. MPI-SDFR-AT-0129
Chaetomium globosum
Chaetomium sp. MPI-CAGE-AT-0009
Chaetomium sp. MPI-CAGE-AT-0009
Dendryphion nanum MPI-CAGE-CH-0243
Cylindrocarpon olidum MPI-CAGE-CH-0241
Halenospora varia MPI-CAGE-AT-0135
Piriformospora indica DSM 11827
Sebacina vermifera MAFF 305830
Fusarium oxysporum f. sp. Lycopersici 4287
Rhexocercosporidium sp. MPI-PUGE-AT-0058
Harpophora oryzae
Magnaporthe grisea
Coprinopsis sp. MPI-PUGE-AT-0042
Microdochium trichocladiopsis MPI-CAGE-CH-0230
Auriculariales sp. MPI-PUGE-AT-0066
Dactylonectria macrodidyma MPI-CAGE-AT-0147
Leptodontidium sp. MPI-SDFR-AT-0119
Ilyonectria europaea MPI-CAGE-AT-0026
Ilyonectria sp. MPI-CAGE-AT-0134
Dactylonectria estremocensis MPI-CAGE-AT-0021
Truncatella angustata MPI-SDFR-AT-0073
Fusarium solani MPI-SDFR-AT-0091
Neonectria ditissima R09/05
Fusarium oxysporum MPI-CAGE-CH-0212
Fusarium verticillioides 7600 v2
Fusarium sp. MPI-SDFR-AT-0072
Fusarium oxysporum MPI-SDFR-AT-0094
Verticillium dahliae MPI-CAGE-AT-0001
Cadophora sp. DSE1049
Leotiomycetes sp. MPI-SDFR-AT-0126
Verticillium alfalfae VaMs.102
Fusarium sp. MPI-CAGE-AA-0113
Fusarium tricinctum MPI-SDFR-AT-0068
Fusarium venenatum MPI-CAGE-CH-0201
Fusarium redolens MPI-CAGE-AT-0023
Fusarium tricinctum MPI-SDFR-AT-0044
Fusarium fujikuroi IMI 58289
Fusarium graminearum
S08A
CE5
CE1
CE8
PL3_2
PL1_4
Lifestyle
SAP
MyM
EF
OMF
PPF
ECM
ERM

Supplementary figure 6: Differential composition in CAZyme, protease, lipase and SSP repertoires according to fungal phylogeny and lifestyle.
a, Distance-based redundancy analyses (dbRDA) of Jaccard distances calculated on the genomic compositions in subfamilies of CAZymes, proteases, lipases, small secreted proteins (SSPs), plant cell wall degrading enzymes (PCWDEs) and fungal cell-wall degrading enzymes (FCWDEs). The left column shows the result of a dbRDA constrained by phylogeny, while the right one shows the results of dbRDA constrained by lifestyle. Both of these factors significantly explain genomic compositions (dbRDA $P < 0.05$; PERMANOVA *JaccardMatrix~Phylogeny+Lifestyle*, $P < 0.05$ - see **Supplementary Data 3** for details). **b,** Double-clustering heatmap of high-loading genes annotated as CAZymes, proteases, lipases or SSPs, which gene counts best segregate lifestyles. S08A: a subfamily S8A secreted serine proteases from proteinase K subfamily. CE: Carbohydrate esterases. PL: Polysaccharide lyases. Colors indicate the fungal lifestyle. Principal components were calculated on total gene counts. High-loading genes were determined based on the first three principal components.
SAP: Saprotrophs, MyM: *A. thaliana* mycobiota members, EF: Endophytic Fungi, OMF: Orchid Mycorrhizal Fungi, PPF: Plant Pathogenic Fungi, ECM: Ectomycorrhiza, ERM: Ericoid Mycorrhiza.

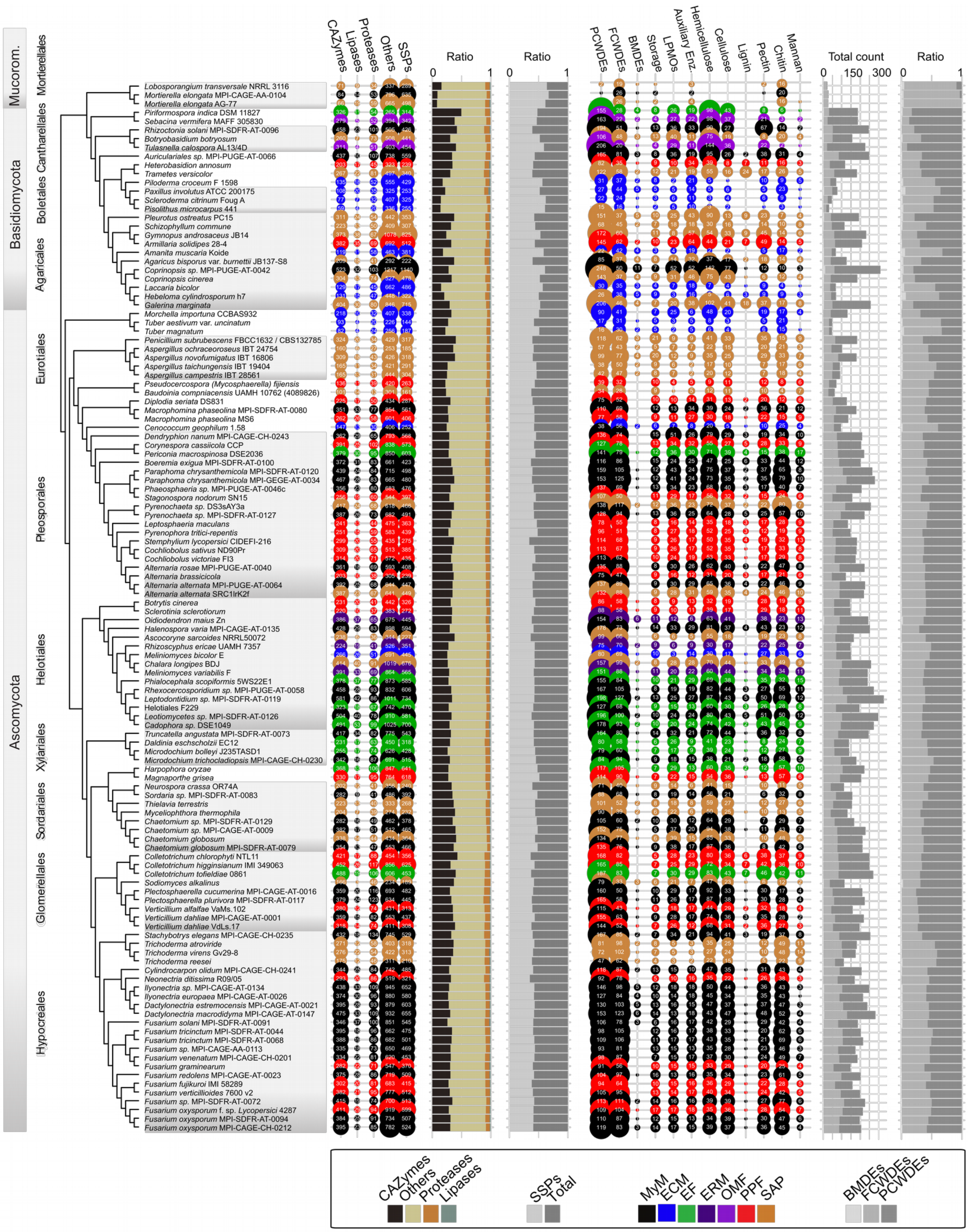
Mucorom.
Basidiomycota
Ascomycota
Mortierellales
Cantharellales
Boletales
Agaricales
Eurotiales
Pleosporales
Helotiales
Xylariales
Sordariales
Glomerellales
Hypocreales
Lobosporangium transversale NRRL 3116
Mortierella elongata MPI-CAGE-AA-0104
Mortierella elongata AG-77
Piriformospora indica DSM 11827
Sebacina vermifera MAFF 305830
Rhizoctonia solani MPI-SDFR-AT-0096
Botryobasidium botryosum
Tulasnella calospora AL13/4D
Auriculariales sp. MPI-PUGE-AT-0066
Heterobasidion annosum
Trametes versicolor
Piloderma croceum F 1598
Paxillus involutus ATCC 200175
Scleroderma citrinum Foug A
Pisolithus microcarpus 441
Pleurotus ostreatus PC15
Schizophyllum commune
Gymnopus androsaceus JB14
Armillaria solidipes 28-4
Amanita muscaria Koide
Agaricus bisporus var. burnettii JB137-S8
Coprinopsis sp. MPI-PUGE-AT-0042
Coprinopsis cinerea
Laccaria bicolor
Hebeloma cylindrosporum h7
Galerina marginata
Morchella importuna CCBAS932
Tuber aestivum var. uncinatum
Tuber magnatum
Penicillium subrubescens FBCC1632 / CBS132785
Aspergillus ochraceoroseus IBT 24754
Aspergillus novofumigatus IBT 16806
Aspergillus taichungensis IBT 19404
Aspergillus campestris IBT 28561
Pseudocercospora (Mycosphaerella) fijiensis
Baudoinia compniacensis UAMH 10762 (4089826)
Diplodia seriata DS831
Macrophomina phaseolina MPI-SDFR-AT-0080
Macrophomina phaseolina MS6
Cenococcum geophilum 1.58
Dendryphion nanum MPI-CAGE-CH-0243
Corynespora cassiicola CCP
Periconia macrospinosa DSE2036
Boeremia exigua MPI-SDFR-AT-0100
Paraphoma chrysanthemicola MPI-SDFR-AT-0120
Paraphoma chrysanthemicola MPI-GEGE-AT-0034
Phaeosphaeria sp. MPI-PUGE-AT-0046c
Stagonospora nodorum SN15
Pyrenochaeta sp. DS3sAY3a
Pyrenochaeta sp. MPI-SDFR-AT-0127
Leptosphaeria maculans
Pyrenophora tritici-repentis
Stemphylium lycopersici CIDEFI-216
Cochliobolus sativus ND90Pr
Cochliobolus victoriae FI3
Alternaria rosae MPI-PUGE-AT-0040
Alternaria brassicicola
Alternaria alternata MPI-PUGE-AT-0064
Alternaria alternata SRC1lrK2f
Botrytis cinerea
Sclerotinia sclerotiorum
Oidiodendron maius Zn
Halenospora varia MPI-CAGE-AT-0135
Ascocoryne sarcoides NRRL50072
Rhizoscyphus ericae UAMH 7357
Meliniomyces bicolor E
Chalara longipes BDJ
Meliniomyces variabilis F
Phialocephala scopiformis 5WS22E1
Rhexocercosporidium sp. MPI-PUGE-AT-0058
Leptodontidium sp. MPI-SDFR-AT-0119
Helotiales F229
Leotiomycetes sp. MPI-SDFR-AT-0126
Cadophora sp. DSE1049
Truncatella angustata MPI-SDFR-AT-0073
Daldinia eschscholzii EC12
Microdochium bolleyi J235TASD1
Microdochium trichocladiopsis MPI-CAGE-CH-0230
Harpophora oryzae
Magnaporthe grisea
Neurospora crassa OR74A
Sordaria sp. MPI-SDFR-AT-0083
Thielavia terrestris
Myceliophthora thermophila
Chaetomium sp. MPI-SDFR-AT-0129
Chaetomium sp. MPI-CAGE-AT-0009
Chaetomium globosum
Chaetomium globosum MPI-SDFR-AT-0079
Colletotrichum chlorophyti NTL11
Colletotrichum higginsianum IMI 349063
Colletotrichum tofieldiae 0861
Sodiomyces alkalinus
Plectosphaerella cucumerina MPI-CAGE-AT-0016
Plectosphaerella plurivora MPI-SDFR-AT-0117
Verticillium alfalfae VaMs.102
Verticillium dahliae MPI-CAGE-AT-0001
Verticillium dahliae VdLs.17
Stachybotrys elegans MPI-CAGE-CH-0235
Trichoderma atroviride
Trichoderma virens Gv29-8
Trichoderma reesei
Cylindrocarpon olidum MPI-CAGE-CH-0241
Neonectria ditissima R09/05
Ilyonectria sp. MPI-CAGE-AT-0134
Ilyonectria europaea MPI-CAGE-AT-0026
Dactylonectria estremocensis MPI-CAGE-AT-0021
Dactylonectria macrodidyma MPI-CAGE-AT-0147
Fusarium solani MPI-SDFR-AT-0091
Fusarium tricinctum MPI-SDFR-AT-0044
Fusarium tricinctum MPI-SDFR-AT-0068
Fusarium sp. MPI-CAGE-AA-0113
Fusarium venenatum MPI-CAGE-CH-0201
Fusarium graminearum
Fusarium redolens MPI-CAGE-AT-0023
Fusarium fujikuroi IMI 58289
Fusarium verticillioides 7600 v2
Fusarium sp. MPI-SDFR-AT-0072
Fusarium oxysporum f. sp. Lycopersici 4287
Fusarium oxysporum MPI-SDFR-AT-0094
Fusarium oxysporum MPI-CAGE-CH-0212
CAZymes
Lipases
Proteases
Others
SSPs
Ratio
0
1
Ratio
0
1
PCWDEs
FCWDEs
BMDEs
Storage
LPMOs
Auxiliary Enz.
Hemicellulose
Cellulose
Lignin
Pectin
Chitin
Mannan
Total count
0
150
300
Ratio
0
1
CAZymes
Others
Proteases
Lipases
SSPs
Total
MyM
ECM
EF
ERM
OMF
PPF
SAP
BMDEs
FCWDEs
PCWDEs

Supplementary figure 7: Descriptions and compositions of predicted fungal secretomes.

The first bubble plot (on the left) shows the number of secreted genes for CAZymes, lipases, proteases, and others (*i.e.*, all secreted proteins not in these first three groups). The group SSPs is a subcategory showing the number of secreted proteins < 300 aa. The size of bubbles corresponds to the number of genes. The fungi are colored according to their ecology. The first bar plots (in the middle) represent the ratio of CAZymes, lipases and proteases, to all secreted proteins (left); and the ratio of SSPs among the entire secretome (right). The second bubble plot (on the right) shows CAZymes grouped according to their functions including plant cell-wall degrading enzymes (PCWDEs) and fungal cell wall degrading enzymes (FCWDEs), peptidoglycans (*i.e.*, bacterial membrane) degrading enzymes (BMDEs), trehalose, starch, glycogen degrading enzymes (Storage), lytic polysaccharide monooxygenase (LPMOs), substrate-specific enzymes for cellulose, hemicellulose, lignin, and pectin (plant cell walls); chitin, glucan, mannan (fungal cell walls). The second bar plots (far right) show the total count of genes including PCWDEs, FCWDEs, and BMDEs (left); and the proportion of PCWDEs, FCWDEs, and BMDEs (right).
MyM: *A. thaliana* mycobiota members, ECM: Ectomycorrhiza, EF: Endophytic Fungi, ERM: Ericoid Mycorrhiza, OMF: Orchid Mycorrhizal Fungi, PPF: Plant Pathogenic Fungi, SAP: Saprotrophs.

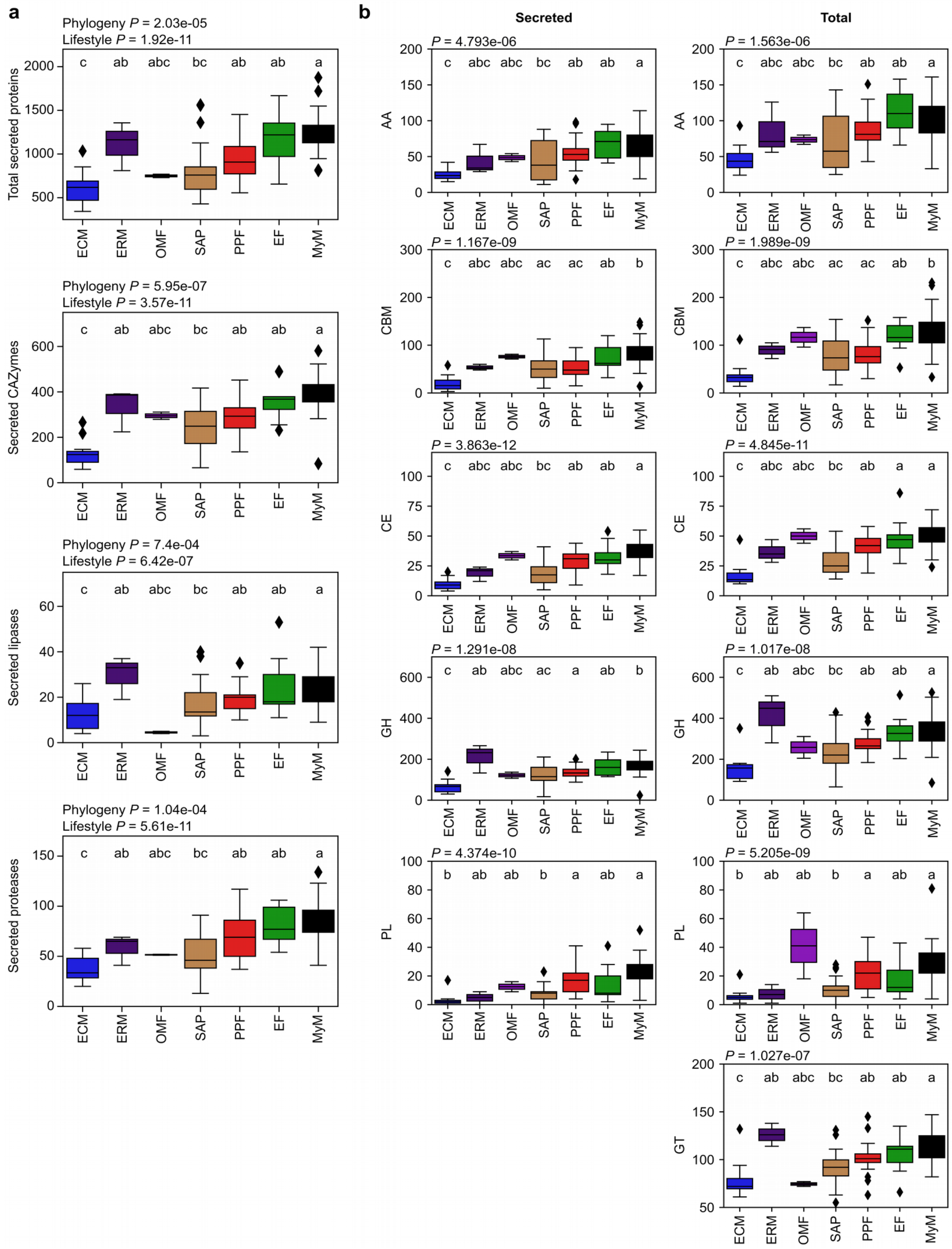
a
Phylogeny P = 2.03e-05
Lifestyle P = 1.92e-11
Total secreted proteins
Phylogeny P = 5.95e-07
Lifestyle P = 3.57e-11
Secreted CAZymes
Phylogeny P = 7.4e-04
Lifestyle P = 6.42e-07
Secreted lipases
Phylogeny P = 1.04e-04
Lifestyle P = 5.61e-11
Secreted proteases
ECM ERM OMF SAP PPF EF MyM
b
Secreted
Total
AA
P = 4.793e-06
P = 1.563e-06
CBM
P = 1.167e-09
P = 1.989e-09
CE
P = 3.863e-12
P = 4.845e-11
GH
P = 1.291e-08
P = 1.017e-08
PL
P = 4.374e-10
P = 5.205e-09
GT
P = 1.027e-07

Supplementary figure 8: Genomic counts of secreted CAZymes (and subfamilies), proteases and lipases across fungal lifestyles.

a, Genomic counts across our comparative genomics data set (n = 120) of total secreted proteins, and secreted CAZymes, lipases and proteases. ANOVA-statistical testing (*Counts~PhylogenyPCs+Lifestyle*, **Methods**) identified both phylogeny and lifestyles as having an effect on genomic contents ($P < 0.05$ - see values on figure); letters result from two-sided post-hoc TukeyHSD testing. **b**, Gene counts across our comparative genomics data set (n = 120) of CAZyme families (AA: Auxiliary Activities, CBM: Carbohydrate-Binding Modules, CE: Carbohydrate Esterases, GH: Glycoside Hydrolases, PL: Polysaccharide Lyases), predicted as secreted (extracellular, left) and total (intra and extracellular, right). Statistical testing with a Kruskal-Wallis test (*Counts~Lifestyle*, $P < 0.05$ - see values on figure) identified lifestyle as having an effect on genome contents. Letters result from post-hoc testing with a two-sided Dunn test.

ECM: Ectomycorrhiza, ERM: Ericoid Mycorrhiza, OMF: Orchid Mycorrhizal Fungi, SAP: Saprotrophs, PPF: Plant Pathogenic Fungi, EF: Endophytic Fungi, MyM: *A. thaliana* mycobiota members. Boxes are delimited by first and third quartiles, central bars show median values, whiskers extend to show the rest of the distribution, but without covering outlier data points (further than 1.5 interquartile range from the quartiles, and marked by lozenges).

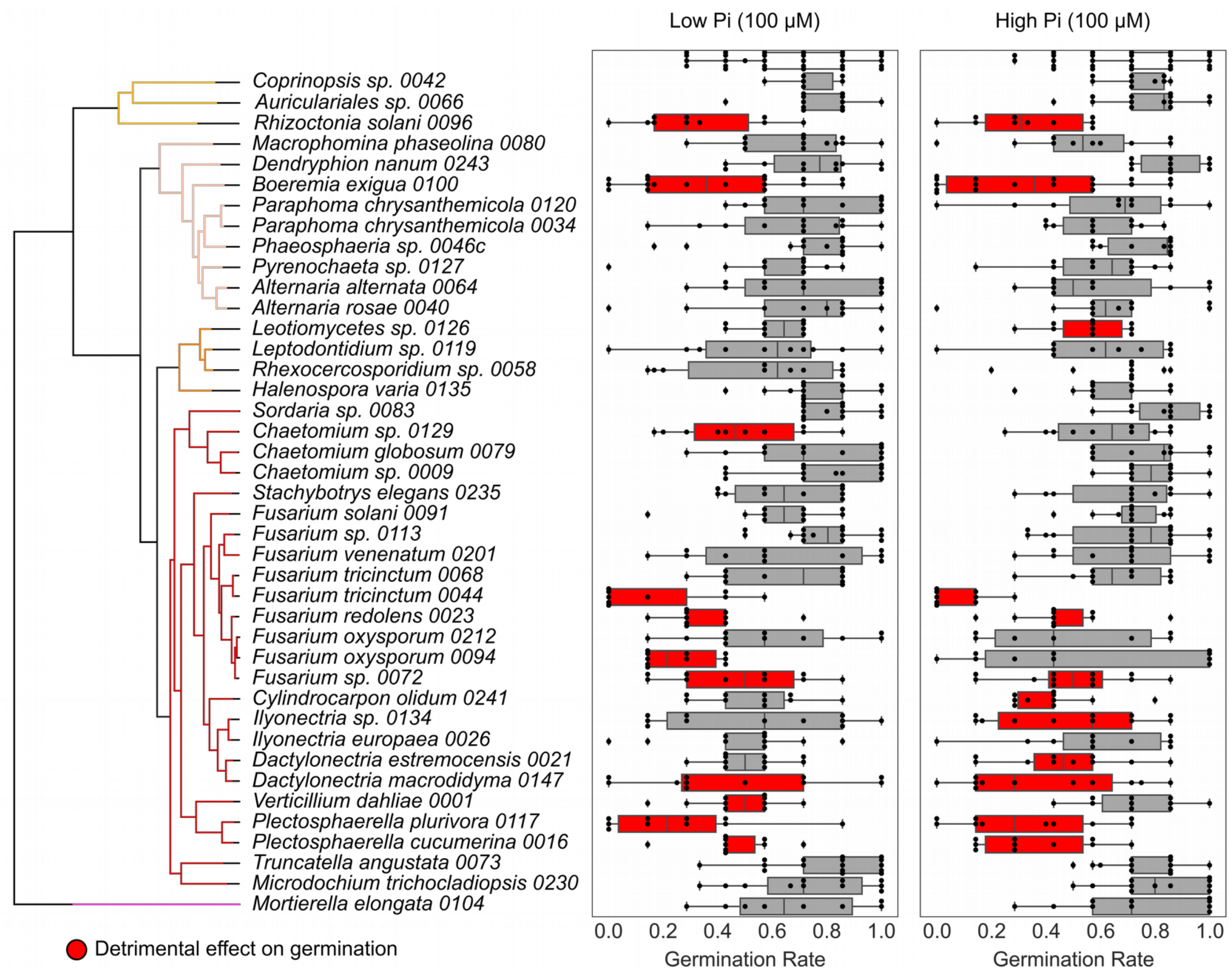

Supplementary figure 9: Fungal effects on *A. thaliana* germination.
Germination rate — proportion of plants that developed over the total number of seeds sowed on each culture plate (n = 6-18) — of *A. thaliana* plants which seeds were inoculated with each of the 41 fungal strains on media containing low and high concentrations of orthophosphate (Pi). Boxes are delimited by first and third quartiles, central bars show median values, whiskers extend to show the rest of the distribution, but without covering outlier data points (further than 1.5 interquartile range from the quartiles). Differential fungal effects on germination rates were tested on both media with Kruskal-Wallis ($P < 10^{-15}$) and beneficial and pathogenic strains were identified by a two-sided Dunn test against mock-treated plants (first row in boxplots).

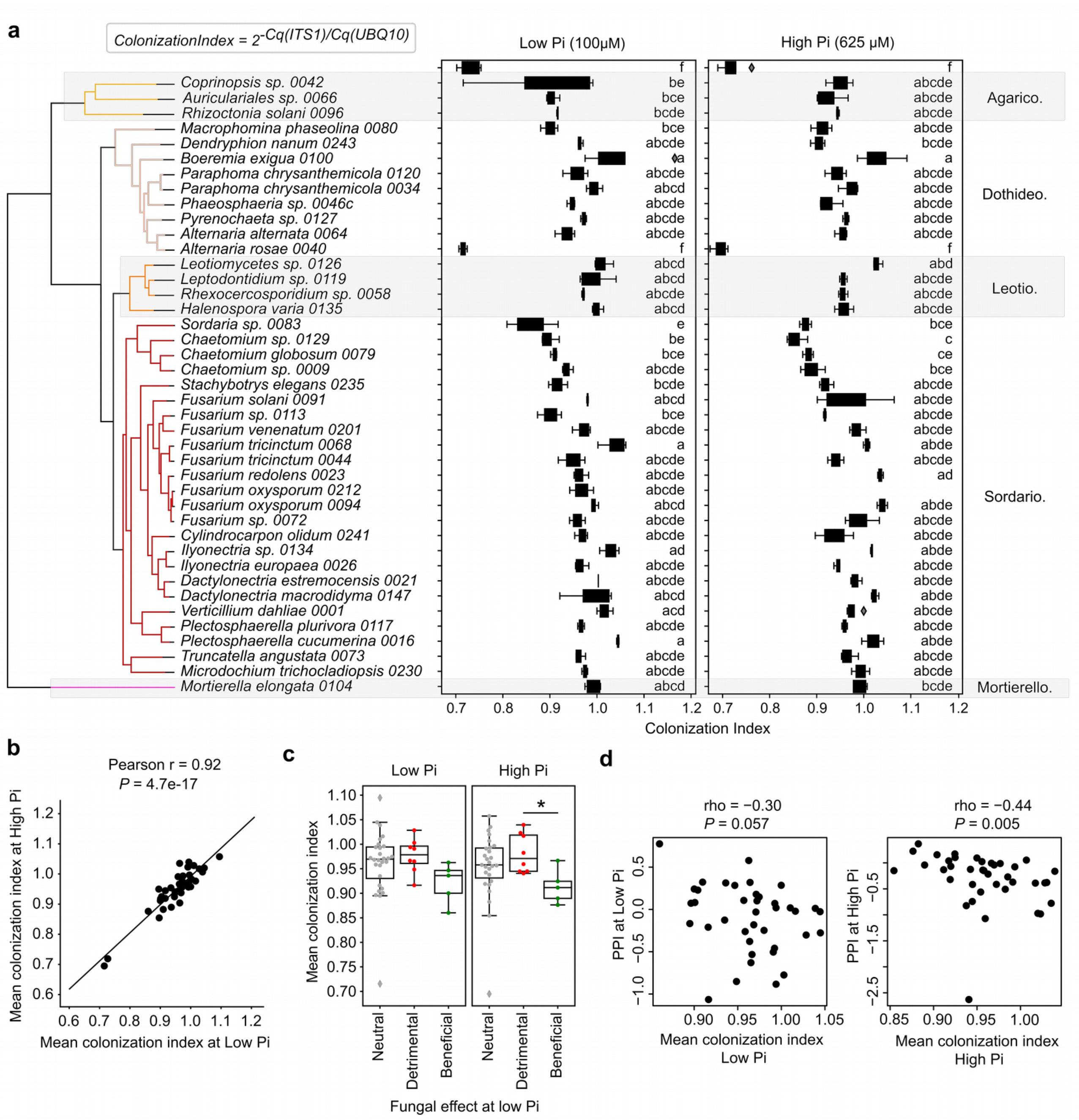

Supplementary figure 10: Fungal colonization of *A. thaliana* roots after 28 days of culture in mono-association.

a, Fungal colonization of plant roots mono-inoculated with different mycobiota members, estimated by quantitative PCR (**Methods**). Boxes are delimited by first and third quartiles, central bars show median values, whiskers extend to show the rest of the distribution, but without covering outlier data points (further than 1.5 interquartile range from the quartiles, and marked by lozenges). Statistical difference across treatments was identified by ANOVA (*ColonizationIndex~Treatment, P* < 1e-17), and two-sided post-hoc testing was performed with TukeyHSD. Colonization was measured in n = 3 root samples per condition. **b**, Pearson correlation (r, *P* < 0.05, n = 41) between mean colonization indexes at low and high Pi concentrations. **c**, Differences in colonization indices between neutral (n = 28), beneficial (n = 5), and detrimental (n = 8) fungi at low Pi. Boxes are delimited by first and third quartiles, central bars show median values, whiskers extend to show the rest of the distribution, but without covering outlier data points (further than 1.5 interquartile range from the quartiles, and marked by lozenges). Significant differences across fungal groups were identified at high Pi, by ANOVA (*P* = 0.0343) and two-sided TukeyHSD tests. (*: adjusted *P* = 0.0297). No significant difference was identified at low Pi

(ANOVA $P = 0.2$) **d**, Correlation between plant performance index and mean colonization index at low Pi (left) and high Pi (right) (n = 41; Spearman's rank correlation rho, $P < 0.05$).

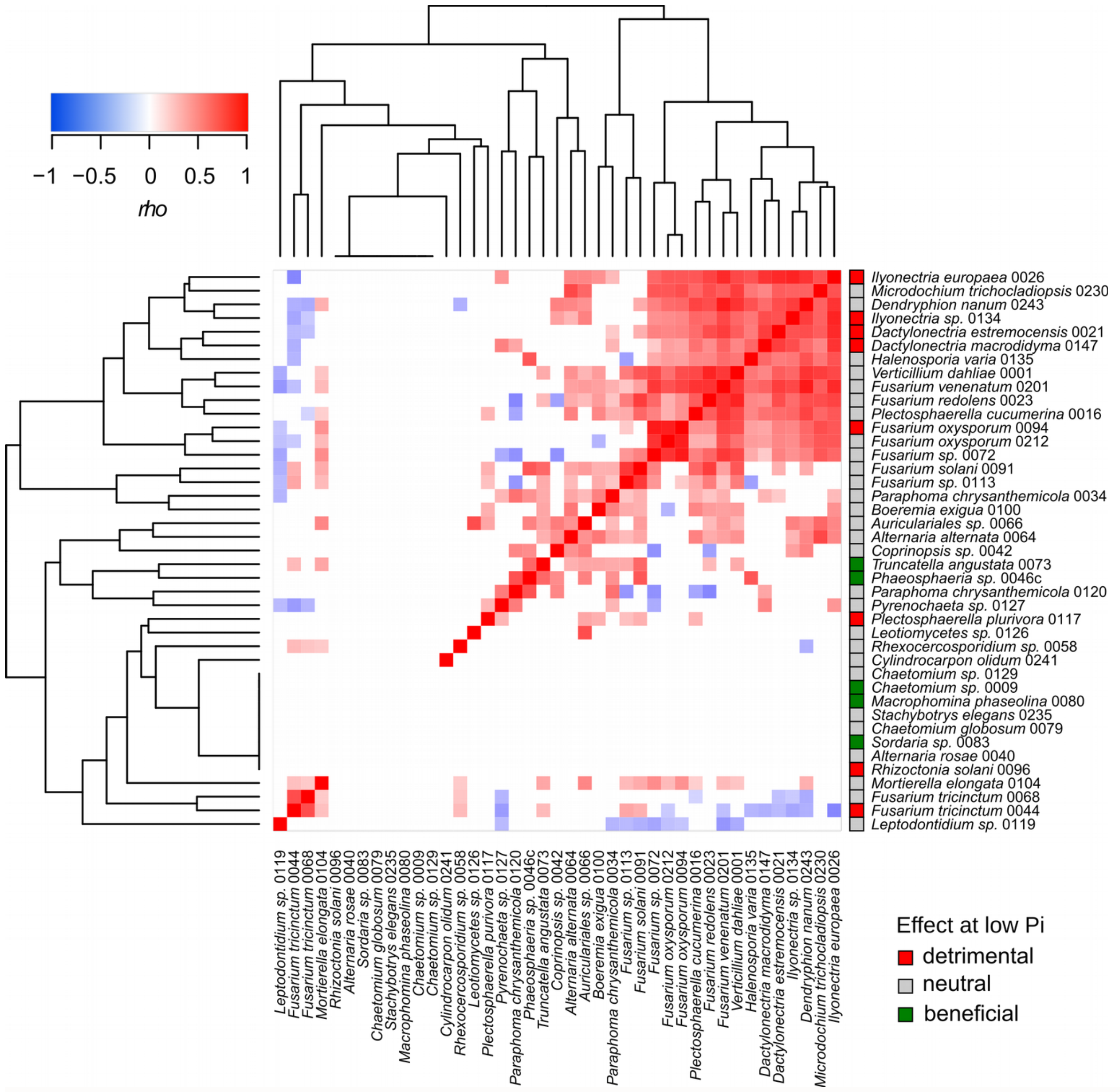

Supplementary figure 11: Correlation matrix comparing the relative abundance profiles of the 41 root mycobiota members in naturally occurring root mycobiomes. Correlation of the 41 fungal taxa relative abundance values across root samples from the European transect data[18]. Spearman's rank correlation (*rho*) was calculated for each fungal pair if these are co-occurring in at least 10 root samples. Only the *rho* values of significant correlations ($P < 0.05$) are plotted. Right to the heatmap, a color-strip indicates if one fungal isolate was identified as having a beneficial, neutral or detrimental effect on plant growth, in mono-association on low Pi agar medium (see Figure 4).

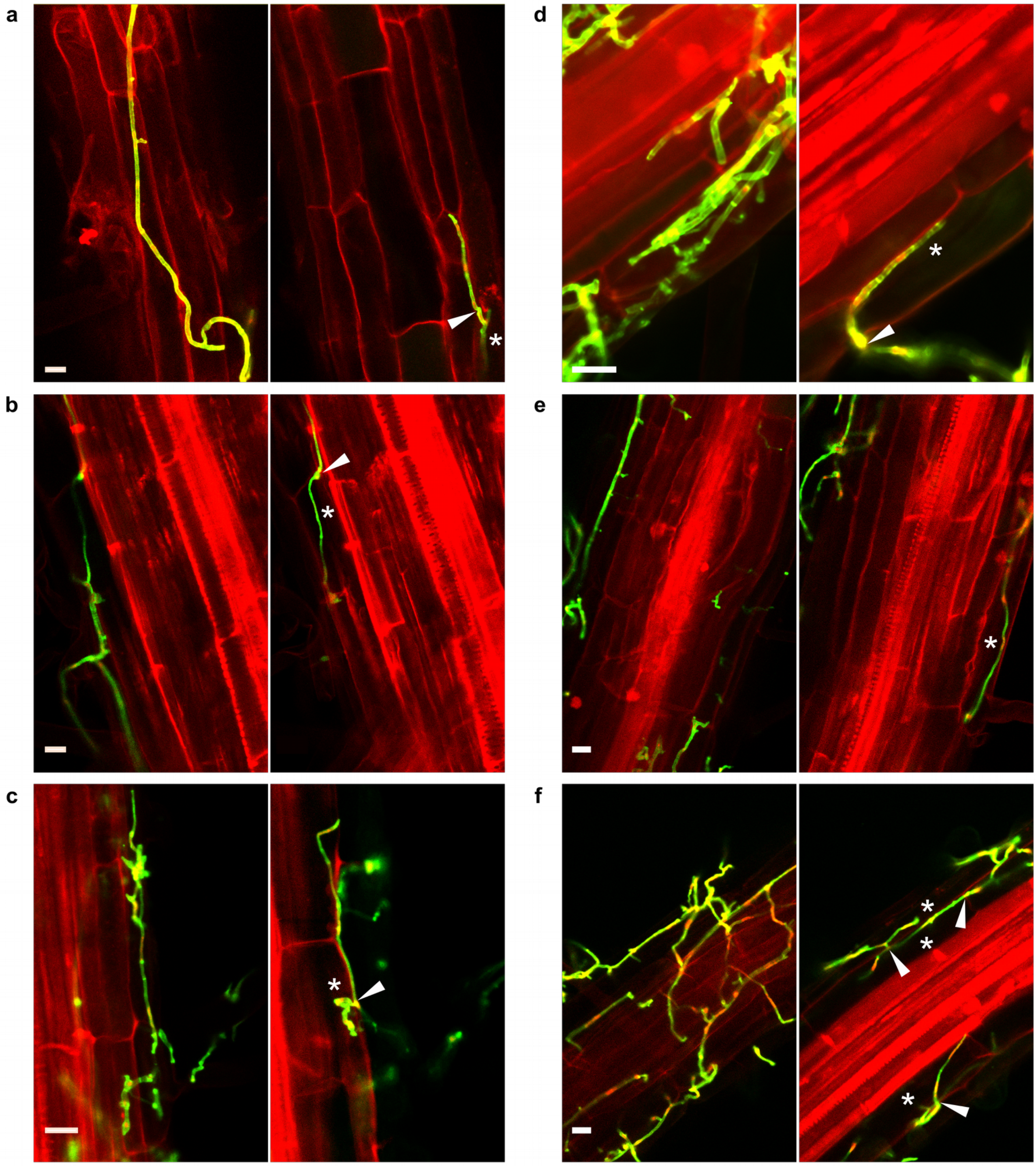

Supplementary figure 12: Confocal imaging of *A. thaliana* root surface and epidermis colonized by six different root mycobiota members.

Roots grown for 4 weeks in mono-association with six diverse fungi, double-stained with Propidium Iodide and Wheat Germ Agglutinin coupled to fluorophore CF®488A (WGA-CF488; Biotium), imaged by confocal microscopy. Left and right picture belong to a single z-stack, respectively focusing on the root surface and the root endosphere where colonization of epidermal cells can be observed. 10µm-scale bars are shown on the left of each panel. Arrows indicate penetration sites and asterisks infected root cells. Similar colonization patterns were observed on 7 different plants in 3 biological replicates. **a,** *Cs = Chaetomium sp. 0009.* **b**, *Mp = Macrophomina phaseolina 0080.* ***c**, Pc= Paraphoma chrysanthemicola 0120.* ***d**, Ps = Phaeosphaeria sp. 0046c.* **e**, *Ta = Truncatella angustata 0073.* **f**, *Hv = Halenospora varia 0135*.

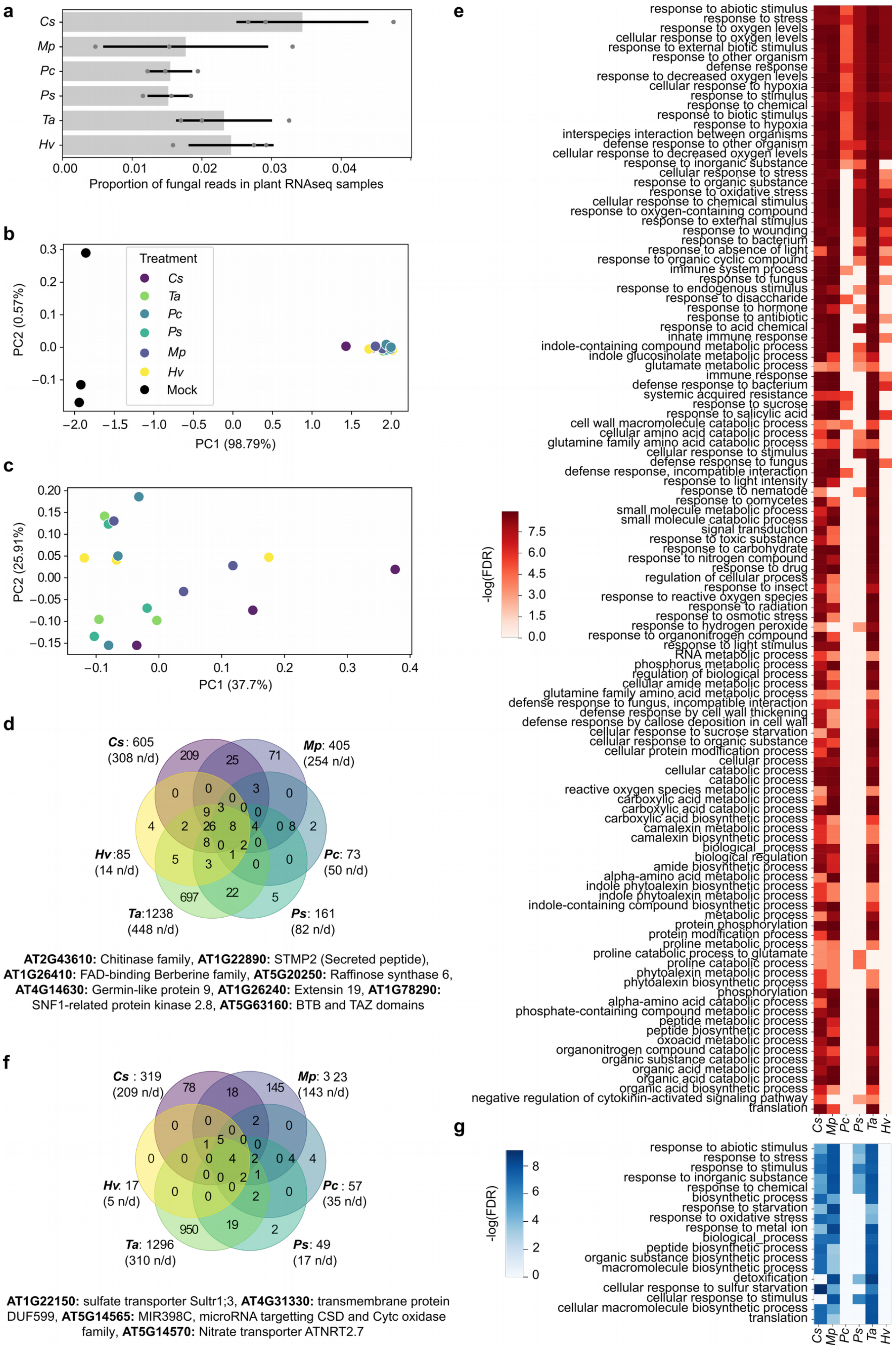
a
Cs
Mp
Pc
Ps
Ta
Hv
0.00 0.01 0.02 0.03 0.04
Proportion of fungal reads in plant RNAseq samples
b
Treatment
Cs
Ta
Pc
Ps
Mp
Hv
Mock
PC2 (0.57%)
PC1 (98.79%)
c
PC2 (25.91%)
PC1 (37.7%)
d
Cs: 605 (308 n/d)
Mp: 405 (254 n/d)
Hv:85 (14 n/d)
Pc: 73 (50 n/d)
Ta:1238 (448 n/d)
Ps: 161 (82 n/d)
AT2G43610: Chitinase family, AT1G22890: STMP2 (Secreted peptide), AT1G26410: FAD-binding Berberine family, AT5G20250: Raffinose synthase 6, AT4G14630: Germin-like protein 9, AT1G26240: Extensin 19, AT1G78290: SNF1-related protein kinase 2.8, AT5G63160: BTB and TAZ domains
e
response to abiotic stimulus
response to stress
response to oxygen levels
cellular response to oxygen levels
response to external biotic stimulus
response to other organism
defense response
response to decreased oxygen levels
cellular response to hypoxia
response to stimulus
response to chemical
response to biotic stimulus
response to hypoxia
interspecies interaction between organisms
defense response to other organism
cellular response to decreased oxygen levels
response to inorganic substance
cellular response to stress
response to organic substance
response to oxidative stress
cellular response to chemical stimulus
response to oxygen-containing compound
response to external stimulus
response to wounding
response to bacterium
response to absence of light
response to organic cyclic compound
immune system process
response to fungus
response to endogenous stimulus
response to disaccharide
response to hormone
response to antibiotic
response to acid chemical
innate immune response
indole-containing compound metabolic process
indole glucosinolate metabolic process
glutamate metabolic process
immune response
defense response to bacterium
systemic acquired resistance
response to sucrose
response to salicylic acid
cell wall macromolecule catabolic process
cellular amino acid catabolic process
glutamine family amino acid catabolic process
cellular response to stimulus
defense response to fungus
defense response, incompatible interaction
response to light intensity
response to nematode
response to oomycetes
small molecule metabolic process
small molecule catabolic process
signal transduction
response to toxic substance
response to carbohydrate
response to nitrogen compound
response to drug
regulation of cellular process
response to insect
response to reactive oxygen species
response to radiation
response to osmotic stress
response to hydrogen peroxide
response to organonitrogen compound
response to light stimulus
RNA metabolic process
phosphorus metabolic process
regulation of biological process
cellular amide metabolic process
glutamine family amino acid metabolic process
defense response to fungus, incompatible interaction
defense response by cell wall thickening
defense response by callose deposition in cell wall
cellular response to sucrose starvation
cellular response to organic substance
cellular protein modification process
cellular process
cellular catabolic process
catabolic process
reactive oxygen species metabolic process
carboxylic acid metabolic process
carboxylic acid catabolic process
carboxylic acid biosynthetic process
camalexin metabolic process
camalexin biosynthetic process
biological_process
biological regulation
amide biosynthetic process
alpha-amino acid metabolic process
indole phytoalexin biosynthetic process
indole phytoalexin metabolic process
indole-containing compound biosynthetic process
metabolic process
protein phosphorylation
protein modification process
proline metabolic process
proline catabolic process to glutamate
proline catabolic process
phytoalexin metabolic process
phytoalexin biosynthetic process
phosphorylation
alpha-amino acid catabolic process
phosphate-containing compound metabolic process
peptide metabolic process
peptide biosynthetic process
oxoacid metabolic process
organonitrogen compound catabolic process
organic substance catabolic process
organic acid metabolic process
organic acid catabolic process
organic acid biosynthetic process
negative regulation of cytokinin-activated signaling pathway
translation
Cs Mp Pc Ps Ta Hv
-log(FDR)
f
Cs : 319 (209 n/d)
Mp: 3 23 (143 n/d)
Hv: 17 (5 n/d)
Pc : 57 (35 n/d)
Ta: 1296 (310 n/d)
Ps: 49 (17 n/d)
AT1G22150: sulfate transporter Sultr1;3, AT4G31330: transmembrane protein DUF599, AT5G14565: MIR398C, microRNA targetting CSD and Cytc oxidase family, AT5G14570: Nitrate transporter ATNRT2.7
g
response to abiotic stimulus
response to stress
response to stimulus
response to inorganic substance
response to chemical
biosynthetic process
response to starvation
response to oxidative stress
response to metal ion
biological_process
peptide biosynthetic process
organic substance biosynthetic process
macromolecule biosynthetic process
detoxification
cellular response to sulfur starvation
cellular response to stimulus
cellular macromolecule biosynthetic process
translation
-log(FDR)

Supplementary figure 13: *A. thaliana* transcriptional reprogramming upon colonization by six different root mycobiota members.

a, Proportion of reads in RNA-Seq samples mapped on fungal genomes. Mean values are shown by the bar plot. Error bars show standard deviation values. n=3 samples per condition, from three independent biological replicates **b**, Principal Component Analysis of Bray-Curtis distances calculated over *A. thaliana* gene read counts. **c**, Principal Component Analysis of Bray-Curtis distances calculated over *A. thaliana* gene read counts, excluding mock-treated samples to reveal sample differences due to the different fungi. *Cs* = *Chaetomium sp. 0009*, *Mp* = *Macrophomina phaseolina* 0080, *Pc* = *Paraphoma chrysantemicola* 0034, *Ps* = *Phaeosphaeria sp.* 0046c, *Ta* = *Truncatella angustata 0073*, *Hv* = *Halenospora varia* 0135. **d**, Venn diagram showing *A. thaliana* commonly over-expressed genes in response to fungal inoculations. Below is the list of genes over-expressed in response to all six fungi. **e**, Independent GO enrichment analyses performed on the *A. thaliana* genes over-expressed in response to each fungus (GOATOOLS[51], FDR < 0.05). **f**, Venn diagram showing *A. thaliana* commonly under-expressed genes in response to fungal inoculations. Below is the list of genes under-expressed in response to all six fungi. **g**, Independent GO enrichment analyses performed on the *A. thaliana* genes under-expressed in response to each fungus (GOATOOLS[51], FDR < 0.05).

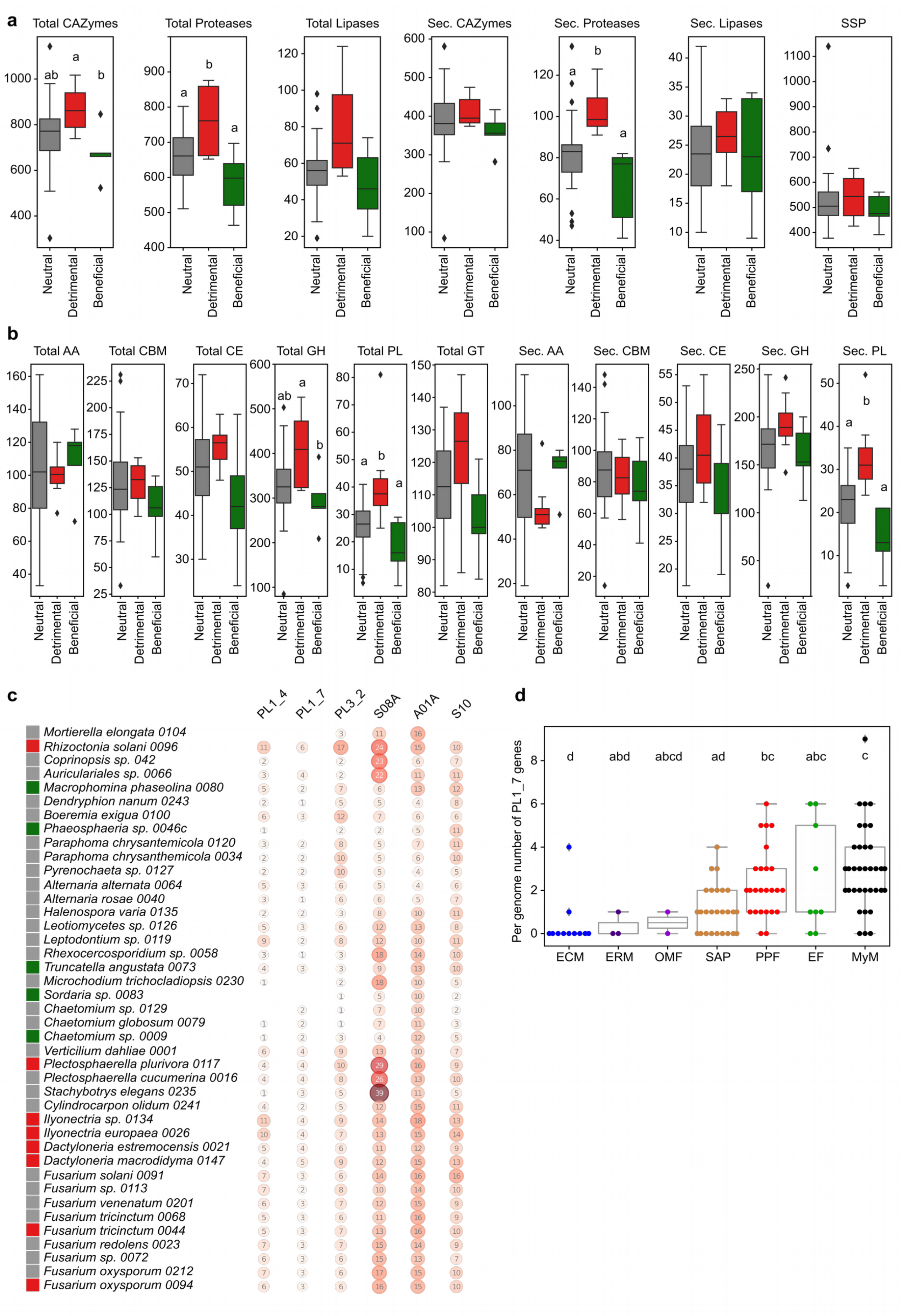
a
Total CAZymes
Total Proteases
Total Lipases
Sec. CAZymes
Sec. Proteases
Sec. Lipases
SSP
Neutral
Detrimental
Beneficial
b
Total AA
Total CBM
Total CE
Total GH
Total PL
Total GT
Sec. AA
Sec. CBM
Sec. CE
Sec. GH
Sec. PL
c
PL1_4
PL1_7
PL3_2
S08A
A01A
S10
Mortierella elongata 0104
Rhizoctonia solani 0096
Coprinopsis sp. 042
Auriculariales sp. 0066
Macrophomina phaseolina 0080
Dendryphion nanum 0243
Boeremia exigua 0100
Phaeosphaeria sp. 0046c
Paraphoma chrysantemicola 0120
Paraphoma chrysanthemicola 0034
Pyrenochaeta sp. 0127
Alternaria alternata 0064
Alternaria rosae 0040
Halenospora varia 0135
Leotiomycetes sp. 0126
Leptodontium sp. 0119
Rhexocercosporidium sp. 0058
Truncatella angustata 0073
Microchodium trichocladiopsis 0230
Sordaria sp. 0083
Chaetomium sp. 0129
Chaetomium globosum 0079
Chaetomium sp. 0009
Verticilium dahliae 0001
Plectosphaerella plurivora 0117
Plectosphaerella cucumerina 0016
Stachybotrys elegans 0235
Cylindrocarpon olidum 0241
Ilyonectria sp. 0134
Ilyonectria europaea 0026
Dactyloneria estremocensis 0021
Dactyloneria macrodidyma 0147
Fusarium solani 0091
Fusarium sp. 0113
Fusarium venenatum 0201
Fusarium tricinctum 0068
Fusarium tricinctum 0044
Fusarium redolens 0023
Fusarium sp. 0072
Fusarium oxysporum 0212
Fusarium oxysporum 0094
d
Per genome number of PL1_7 genes
ECM
ERM
OMF
SAP
PPF
EF
MyM

Supplementary figure 14: Genomic signatures in polysaccharide lyase repertoires explain lifestyle differentiation among root mycobiota members.

a, Distribution of genes encoding secreted and total CAZymes, lipases, proteases and SSPs, in the genomes of the 41 mycobiota members. **b,** Distribution of genes inside each CAZyme family. In **a** and **b**, boxes are delimited by first and third quartiles, central bars show median values, whiskers extend to show the rest of the distribution, but without covering outlier data points (further than 1.5 interquartile range from the quartiles, and marked by lozenges). The different letters indicate significant difference (FDR < 0.05; Kruskal-Wallis and two-sided Dunn test). Beneficial ($n = 5$), neutral ($n = 26$), pathogenic ($n = 10$). **c,** Key secreted protein coding genes discriminating fungal lifestyles of 41 endophytic fungi. Three fungal effects on plant growth (in mono-association on low Pi medium, see Figure 4) are depicted in different colors (green: beneficial, red: detrimental, grey: neutral). The selected genes coding for secreted polysaccharide lyases (PLs) and proteases discriminate between pathogenic, neutral, and beneficial fungi. Fungal taxa are displayed according to the phylogenetic order. Bubbles with numbers contain the number of genes. **d,** Comparative genomics of the PL1_7 CAZyme subfamily, showing the number of PL1_7 genes in the genomes (n=120) associated to different lifestyles. Boxes are delimited by first and third quartiles, central bars show median values, whiskers extend to show the rest of the distribution, but without covering outlier data points (further than 1.5 interquartile range from the quartiles). Statistics were performed using an ANOVA test (*Counts~Lifestyle*, $P = 1.07e\text{-}06$) and a two-sided TukeyHSD post-hoc test (adjusted $P < 0.05$).
