# Supplementary material for: Genetic determinants of endophytism in the Arabidopsis root mycobiome
Source: Nat Commun. 2021 Dec 10;12:7227. doi: 10.1038/s41467-021-27479-y (PMC8664821; doi:10.1038/s41467-021-27479-y)
Supplement: Supplementary file 3 — Description of Additional Supplementary Files [file 41467_2021_27479_MOESM3_ESM.pdf]

Description of Additional Supplementary Files

File Name: Supplementary Data 1
Description: **Description of the 41 newly-sequenced strains of Arabidopsis thaliana root mycobiota members.** This table provides information regarding the phylogeny of the 41 strains (phylum, class, order, species), as well as information regarding the isolation of these strains (host, location) and statistics regarding the genome assemblies (Assembly size, number of contigs, L50, N50), and number of predicted genes. Genbank bioproject, biosample and accession IDs are provided in the last three columns of the table.

File Name: Supplementary Data 2
Description: **Description of the comparative genomics dataset, comprising our 41 newly-sequenced strains and 79 published fungal genomes.** This table provides information regarding the phylogeny of the 120 strains (phylum, class, order, strain), as well as information regarding the isolation of these strains (host, location, niche), statistics about the genome assemblies (Assembly size, number of contigs, L50, N50), together with publication IDs (PMID) and URL from which the genomes were downloaded. It also shows the lifestyle attributed to each fungal strain (Assigned lifestyle) and the FunGuild[40] description of the associated strain or genus (Guild FG, Trophic mode FG)

File Name: Supplementary Data 3
Description: **Results of PERMANOVA analyses testing the effect of phylogeny and lifestyle on genomic compositions in gene repertoires.** This table provides the detailed results of independent PERMANOVA analyses testing the effect of phylogeny and lifestyle on Jaccard distance matrices reflecting the genomic compositions in each gene category of interest. Tested factors are the first four Principal Components (PC) of a phylogenetic PCA, fungal lifestyle, and the interaction of each phylogenetic PC with fungal lifestyle.

File Name: Supplementary Data 4
Description: **Description of the 84 orthogroups segregating endophytes and mycobiota members from other fungi.** a) This table provides information regarding the 84 gene families that best segregate endophytes and mycobiota members from others, according to our SVM-RFE classifier ($R2=0.8$), including enrichment/depletion scores in the fungi of interest (Enrichment in EF+MyM) and associated ANOVA P-values (FDR), support vector coefficients in the classifier (SVM coefficients), representative sequence of the family, information about functional annotation (curated description, curated group) and associated COG family used for co-expression analysis.
b) This table provides the results of a GO enrichment analysis performed with GOATOOLS [51] on the 84 orthogroups determinant for endophytism. GOATOOLS performs a two-sided Fisher's exact test. Correction of p-values into FDR was performed using the Benjamini-Hochberg method.
c) Coexpression scores of the 84 gene families in fungal transcriptomic data sets, according to STRING-db[52]**.**

File Name: Supplementary Data 5
Description: **Differential fungal gene expression in planta vs. on medium.** These tables provide read mapping statistics (RPKM values in control samples and in planta test samples) differential expression statistics (baseMean, log2FoldChange, lfcSE, pvalue, padj) from DESeq2 [55], together with functional annotation information (SSP, CAZyme IDs and descriptions, MEROPS Protease IDs and descriptions, Lipase IDs, KOG IDs and descriptions, EC IDs and descriptions, InterPro IDs and descriptions, GO IDs and descriptions. Statistical testing for differential expression was performed by a two-sided Wald test as implemented in DESeq2. Correction of p-values into adjusted p-values (padj) was performed with the DESeq2 built-in method.

5a) *Chaetomium sp.* MPI-CAGE-AT-0009 (Cs)
5b) *Macrophomina phaseolina* MPI-SDFR-AT-0080 (Mp)
5c) *Paraphoma chrysanthemicola* MPI-GEGE-AT-0034 (Pc)
5d) *Phaeosphaeria sp.* MPI-PUGE-AT-0046c (Ps)
5e) *Truncatella angustata* MPI-SDFR-AT-0073 (Ta)
5f) *Halenospora varia* MPI-CAGE-AT-0135 (Hv)

File Name: Supplementary Data 6
Description: **Differential *Arabidopsis thaliana* gene expression inoculated with fungi vs. mock-treated.** These tables provide read mapping statistics (RPKM values in control mock-treated samples and inoculated test samples) differential expression statistics (baseMean, log2FoldChange, lfcSE, pvalue, padj) from DESeq2 [55], and short gene descriptions from TAIR10.
Statistical testing for differential expression was performed by a two-sided Wald test as implemented in DESeq2. Correction of p-values into adjusted p-values (padj) was performed with the DESeq2 built-in method.

6a) *A. thaliana* inoculated with *Chaetomium sp.* MPI-CAGE-AT-0009 (Cs)
6b) *A. thaliana* inoculated with *Macrophomina phaseolina* MPI-SDFR-AT-0080 (Mp)
6c) *A. thaliana* inoculated with *Paraphoma chrysanthemicola* MPI-GEGE-AT-0034 (Pc)
6d) *A. thaliana* inoculated with *Phaeosphaeria sp*. MPI-PUGE-AT-0046c (Ps)
6e) *A. thaliana* inoculated with *Truncatella angustata* MPI-SDFR-AT-0073 (Ta)
6f) *A. thaliana* inoculated with *Halenospora varia* MPI-CAGE-AT-0135 (Hv)

File Name: Supplementary Data 7
Description: **Description of the 11 orthogroups segregating detrimental mycobiota members from others.** This table provides information regarding the 11 gene families that best segregate detrimental mycobiota members from neutral and beneficial ones, according to our SVM-RFE classifier (R2=0.88), including enrichment/depletion scores in the fungi of interest (Enrichment in detrimental fungi) and associated ANOVA P-values (FDR), support vector coefficients in the classifier (SVM coefficients), representative sequence of the family and functional annotation (curated description).
