# Supplementary material for: Genetic determinants of endophytism in the Arabidopsis root mycobiome
Source: Nat Commun. 2021 Dec 10;12:7227. doi: 10.1038/s41467-021-27479-y (PMC8664821; doi:10.1038/s41467-021-27479-y)
Supplement: Supplementary file 11 — Reporting Summary [file 41467_2021_27479_MOESM11_ESM.pdf]

nature portfolio

Corresponding author(s): Stéphane Hacquard
Last updated by author(s): Nov 3, 2021

Reporting Summary

Nature Portfolio wishes to improve the reproducibility of the work that we publish. This form provides structure for consistency and transparency in reporting. For further information on Nature Portfolio policies, see our Editorial Policies and the Editorial Policy Checklist.

Statistics

For all statistical analyses, confirm that the following items are present in the figure legend, table legend, main text, or Methods section.

n/a	Confirmed	
☐	☒	The exact sample size (*n*) for each experimental group/condition, given as a discrete number and unit of measurement
☐	☒	A statement on whether measurements were taken from distinct samples or whether the same sample was measured repeatedly
☐	☒	The statistical test(s) used AND whether they are one- or two-sided<br>*Only common tests should be described solely by name; describe more complex techniques in the Methods section.*
☐	☒	A description of all covariates tested
☐	☒	A description of any assumptions or corrections, such as tests of normality and adjustment for multiple comparisons
☐	☒	A full description of the statistical parameters including central tendency (e.g. means) or other basic estimates (e.g. regression coefficient) AND variation (e.g. standard deviation) or associated estimates of uncertainty (e.g. confidence intervals)
☐	☒	For null hypothesis testing, the test statistic (e.g. *F*, *t*, *r*) with confidence intervals, effect sizes, degrees of freedom and *P* value noted<br>*Give P values as exact values whenever suitable.*
☒	☐	For Bayesian analysis, information on the choice of priors and Markov chain Monte Carlo settings
☒	☐	For hierarchical and complex designs, identification of the appropriate level for tests and full reporting of outcomes
☐	☒	Estimates of effect sizes (e.g. Cohen's *d*, Pearson's *r*), indicating how they were calculated

Our web collection on statistics for biologists contains articles on many of the points above.

Software and code

Policy information about availability of computer code

Data collection	Our confocal microscopy images were collected with the software ZEN v.2.3 SP1 by ZEISS.
Data analysis	Data analyses and statistical testing were performed using languages Python v3.7.3 and R v3.5.1 (except for transcriptomic analyses, for which R v3.6.1 was used). All scripts were deposited at https://github.com/fantin-mesny/Scripts-from-Mesny-et-al.-2021 [97].<br>Other software used for data analysis are: USEARCH v10.0.240[74] (for ITS sequence comparison to published datasets); Falcon v0.7.3[75], Trinity v2.1.1 [76], the JGI annotation pipeline[77] and FGENESH v8.8.0[79] for genome assembly and annotation; OrthoFinder v2.2.7[46] for orthology prediction; GLOOME gainLoss.VR01.266[80], Count v10.04[47] and Sklearn v0.20.3[81] for ancestral lifestyle prediction; BUSCO v3.0.2 [82], TINGO[83] and PRINGO[32] for genomic features annotation and Vegan v2.5-7 and RVAideMemoire v0.9-77 R packages for their analysis; Sklearn v0.20.3[81], FAMSA v1.6.1[88], HMMER v3.2.1[89], GOATOOLS v1.0.3[51] and Phylolm v.2.6.2[50] for identifying genomic determinants of endophytism or of detrimental effects and analysing them; Cytoscape v3.7.2[90] with ClusterMaker2 v1.3.1[91] to cluster gene families according to their co-expression; and finally Trimmomatic v0.38[94], HiSat2 v2.2.0[54], featureCounts v2.0.0[95], DESeq2 v1.24.0[55], apeglm v1.6.0[96] and GOATOOLS v1.0.3[51] for transcriptomics.<br>R library DescTools v0.99.28 was used for non-parametric statistical testing

For manuscripts utilizing custom algorithms or software that are central to the research but not yet described in published literature, software must be made available to editors and reviewers. We strongly encourage code deposition in a community repository (e.g. GitHub). See the Nature Portfolio guidelines for submitting code & software for further information.

Data

Policy information about availability of data

All manuscripts must include a data availability statement. This statement should provide the following information, where applicable:

- Accession codes, unique identifiers, or web links for publicly available datasets
- A description of any restrictions on data availability
- For clinical datasets or third party data, please ensure that the statement adheres to our policy

Raw and processed genome sequencing data are available at MycoCosm (https://mycocosm.jgi.doe.gov/mycocosm/home). In addition, the newly sequenced genome assemblies and annotations have been deposited to GenBank with the following Bioprojects numbers: PRJNA371205, PRJNA347188, PRJNA441695, PRJNA370201, PRJNA571620, PRJNA370120, PRJNA347200, PRJNA371203, PRJNA370196, PRJNA500112, PRJNA370194, PRJNA455444, PRJNA370199, PRJNA347190, PRJNA455442, PRJNA347185, PRJNA370198, PRJNA347189, PRJNA455443, PRJNA500113, PRJNA347186, PRJNA347191, PRJNA370195, PRJNA370119, PRJNA347187, PRJNA500111, PRJNA347192, PRJNA347193, PRJNA538399, PRJNA459235, PRJNA347194, PRJNA371204, PRJNA570880, PRJNA347196, PRJNA347195, PRJNA371202, PRJNA370118, PRJNA370200, PRJNA347197, PRJNA519173, PRJNA370197. Raw transcriptomic data used in our differential gene expression analysis are available at Gene Expression Omnibus: GSE169629. We referred to three online databases for analysis: UNITE (https://unite.ut.ee, version February 2021), GlobalFungi (https://globalfungi.com, version August 2020) and String-db (https://globalfungi.com/, version August 2020). The processed transcriptomic data are also available in this GEO entry. The plant phenotypic data and fungal colonization values are provided in the Source Data file.

Field-specific reporting

Please select the one below that is the best fit for your research. If you are not sure, read the appropriate sections before making your selection.

☒ Life sciences ☐ Behavioural & social sciences ☐ Ecological, evolutionary & environmental sciences

For a reference copy of the document with all sections, see nature.com/documents/nr-reporting-summary-flat.pdf

Life sciences study design

All studies must disclose on these points even when the disclosure is negative.

Sample size	Statistical methods were not used to predetermine sample size. Sample size was decided using preliminary testing for experimental variability, to ensure confident statistical analysis. We took care of producing large replicate numbers per condition (usually n ≥ 9 samples per condition, except for qPCR colonization measurements where n=3), with three or more fully independent biological replicates were performed.<br>In plant-fungi interaction experiments, we prepared 4 plates per condition in each of our 3 independent biological replicates. Each plate contained 7 seeds and was used to calculate a single plant performance. These plant performance indices represent therefore the average effect of a fungus on 7 plants.<br>To generate RNAseq data, we performed three independent replicates (1 plate containing 7 plants per condition) resulting in three RNAseq read samples per condition.<br>To analyze the fungal colonization of plants, the roots of at least 7 inoculated plants from three different replicates were observed by confocal microscopy.
Data exclusions	A low number of RNAseq reads were trimmed or discarded with the software Trimmomatic as their quality did not encounter minimum requirements for further processing.
Replication	All experiments were performed at least three independent times, so data reflect biology more than experimental bias. All findings from replicate experiments were consistent and allowed meaningful statistical analyses.
Randomization	Cultures of each fungal strains were inoculated into plants at random without note of their identity. Each replicate plate of each condition of the experiment was distributed at random into the growth chamber, and randomly shuffled twice a week to minimize location-based effects. Samples were collected randomly for each condition in all experiments.
Blinding	During sample harvesting and measuring shoot fresh weight, no procedure was taken to blind experimenters to the different conditions. However, plates were only labeled with a fungal strain number, and the experimenters did not know the strain name associated to each number. An automatic balance was used to record shoot fresh weight in a data sheet, without interference of the experimenters.<br><br>Harvested tissues were stored in numbered tubes without sample condition or identity labels. The sample metadata was stored in a separate file. Thereby, during the processing of samples for fungal effects on plant growth (including experiments with knock-in lines), RNASeq analysis, and microscopy analysis of fungal colonization, experimenters did not know the identity of the samples.

Reporting for specific materials, systems and methods

We require information from authors about some types of materials, experimental systems and methods used in many studies. Here, indicate whether each material, system or method listed is relevant to your study. If you are not sure if a list item applies to your research, read the appropriate section before selecting a response.

Materials & experimental systems

n/a	Involved in the study
☒	☐ Antibodies
☒	☐ Eukaryotic cell lines
☒	☐ Palaeontology and archaeology
☒	☐ Animals and other organisms
☒	☐ Human research participants
☒	☐ Clinical data
☒	☐ Dual use research of concern

Methods

n/a	Involved in the study
☒	☐ ChIP-seq
☒	☐ Flow cytometry
☒	☐ MRI-based neuroimaging
